# Association of Geriatric Nutritional Risk Index With Cardiovascular and All-Cause Mortality Among US Elderly Adults With Diabetic Nephropathy

**DOI:** 10.1155/jdr/2746706

**Published:** 2025-10-23

**Authors:** Chun Sun, Kun Gao, Keyun Liu

**Affiliations:** Department of Interventional Radiology, Beijing Chaoyang Hospital, Capital Medical University, Beijing, China

**Keywords:** diabetic nephropathy, elderly adults, Geriatric Nutritional Risk Index, NHANES

## Abstract

**Objectives:**

This study examines the association between the Geriatric Nutritional Risk Index (GNRI) and all-cause and cardiovascular disease (CVD) mortality in elderly diabetic nephropathy (DN) patients in the United States.

**Methods:**

Participants were categorized into three GNRI-based groups: (1) moderate to severe malnutrition (M/S) risk (< 92), (2) low risk (92–98), and (3) no risk (> 98). The primary outcomes were all-cause and CVD mortality. Cox proportional hazards regression and competing risk analysis were used to estimate hazard ratios (HRs) and 95% confidence intervals (CIs).

**Results:**

Among 1790 DN patients (mean age 71.24 ± 7.17 years; weighted 55.0% male, 45.0% female), over 11,724 person-years (mean follow-up 6.56 ± 4.59 years), 274 (15.3%) died from CVD and 914 (51.1%) from all causes. The M/S risk group had a 143% increased all-cause mortality risk (HR 2.43; 95% CI, 1.25–5.80), while the low risk group had a 56% increased risk (HR 1.56; 95% CI, 1.19–2.38), compared to the no risk group. No significant correlation was observed between GNRI and CVD mortality. In gender-specific analysis, the M/S risk group had a higher all-cause mortality risk in males (HR 2.50; 95% CI, 1.04–5.99) than in females (HR 1.62; 95% CI, 0.83–3.15).

**Conclusions and Implications:**

A lower GNRI is associated with higher all-cause mortality in elderly DN patients, particularly in males, but not with CVD mortality. GNRI may be a useful prognostic tool for mortality risk assessment in this population.

## 1. Introduction

Diabetic nephropathy (DN), a prevalent complication among diabetic patients, affects up to 40% of this demographic, positioning it as a principal contributor to chronic kidney disease worldwide. This condition is particularly prevalent among individuals aged 60 and above, with more than 25% affected—a figure that continues to grow with the aging of the population [1]. Elevated blood glucose levels are a primary pathogenic factor in elderly patients with DN, leading to renal damage. This damage progressively impairs renal function, evidenced by proteinuria and, ultimately, uremia, which diminishes overall nutritional status [2]. Additional factors such as hypertension, dyslipidemia, inflammatory responses, and genetic predispositions are intricately linked with the onset and progression of this condition [3, 4]. Chronic DN may lead to hypertension, anemia, cardiovascular diseases (CVDs), and, in extreme cases, renal failure. This progression imposes substantial burdens on patients, their families, and society, significantly affecting quality of life and economic stability [5].

Elderly individuals are particularly vulnerable to malnutrition due to physiological aging, chronic diseases, dietary habits, and metabolic changes, which can lead to reduced nutrient intake and absorption. The Geriatric Nutritional Risk Index (GNRI) is a widely utilized tool to assess nutritional status and associated health risks in the elderly, relying on metrics such as weight changes and serum albumin levels. This indicator quantifies the nutritional risk of the elderly, thereby providing important insights into their overall health status [6]. Research has linked low GNRI scores to a range of adverse outcomes, including fractures, CVD, idiopathic pulmonary fibrosis, and poor stroke prognosis in elderly patients [7–10]. A lower GNRI value typically indicates malnutrition or chronic illness, which may lead to reduced immune function, diminished recovery ability, and a decline in quality of life. Given the elderly population's extreme sensitivity to nutritional status, monitoring the GNRI can help clinicians promptly identify potential issues and develop personalized interventions to improve patients' health outcomes. The GNRI is not only an important tool for nutritional assessment in elderly patients but also a critical indicator for predicting their clinical outcomes. It holds significant importance in enhancing the quality of life and health levels of elderly patients. In the context of DN, malnutrition emerges as a critical risk factor that impairs glycemic control, diminishes immune function, and leads to muscle wasting, collectively exacerbating renal burden and accelerating disease progression [11]. Additionally, malnutrition can intensify inflammatory responses and endothelial dysfunction, further hastening the progression of DN [12].

Owing to its ease of use and widespread clinical adoption, the GNRI has been extensively explored across various medical conditions. Research highlights its significant correlation with diabetic retinopathy and its value as an independent predictor of mortality among diabetic foot ulcer patients undergoing amputation [13, 14]. Nonetheless, research on GNRI in the context of DN remains scant. Patients with DN often suffer from chronic metabolic disturbances, leading to systemic health complications and increased mortality from heart failure, coronary artery disease, or infections [15]. Moreover, elevated GNRI risks are linked with the progression of atherosclerosis, hypertension, and CVD among the elderly, adversely affecting immune and organ functions. Additionally, there are studies connecting GNRI with retinopathy in Type 2 diabetes patients [16–18]. Despite these associations, there remains a gap in the literature regarding the impact of GNRI on CVD mortality and all-cause mortality in elderly patients with DN.

Therefore, this study employs data from the National Health and Nutrition Examination Survey (NHANES) spanning 1999–2018 to evaluate the relationship between the GNRI and risks of CVD mortality and all-cause mortality in elderly patients with DN. The analysis controls for a range of demographic, health, and biochemical factors to isolate the impact of GNRI on these outcomes.

## 2. Methods

### 2.1. Study Population

The dataset for this research was sourced from the NHANES database, which is publicly available on the National Center for Health Statistics (NCHS) website. Operated by the NCHS, NHANES aims to evaluate the health and nutritional status of the US population and associated health risk factors. Initiated in 1999, the survey was conducted biennially, with each cycle sampling approximately 10,000 individuals across diverse age groups, genders, races/ethnicities, and geographic regions throughout the United States. The NHANES dataset is multidimensional, encompassing data on health status, nutritional intake, lifestyle, and healthcare service utilization, among other facets. Its data quality and reliability are highly esteemed. The National Center has approved NHANES for the Health Statistics Ethics Review Board. Given that all data is deidentified, informed consent is not required from the patient. Our study adheres to the Strengthening the Reporting of Observational Studies in Epidemiology (STROBE) guidelines [19].

Our investigation encompassed 10 survey cycles spanning from 1999 to 2018, examining the association between the GNRI and both CVD and all-cause mortality in elderly DN patients. Consequently, we established the following exclusion criteria: (1) age below 60 years; (2) uncertain diabetes status, indicated by absent data on fasting blood glucose, glycated hemoglobin, or self-reported diabetes; (3) unconfirmed renal insufficiency due to missing information on urine albumin/creatinine ratio (UACR), blood creatinine, serum albumin, height, or weight; (4) incomplete GNRI data; and (5) absence of essential follow-up data, including mortality outcomes. For covariates with 20%–30% missing data, we used the K-M random forest imputation method to fill in the data, and covariates with more than 30% missing data were deleted.

### 2.2. Definition of GNRI

The GNRI is calculated using the following formula derived from prior studies:

 $$\begin{array}{c} \text{GNRI}=\left[1.489\times \text{serum albumin}\left(\text{g}/\text{L}\right)\right]+\left(41.7\times \text{weight}\left(\text{kg}\right)/\text{ideal weight}\left(\text{kg}\right)\right). \end{array}$$

The ideal weight is calculated using the following equation:

 $$\begin{array}{c} \text{ideal weight}=22\times \text{height}{\left(\text{m}\right)}^{2}. \end{array}$$

If a patient's actual weight surpasses their ideal weight, the weight-to-ideal weight ratio is capped at 1 [20, 21]. Based on GNRI values, patients are classified based on the following thresholds: (1) moderate to severe malnutrition (M/S) risk: < 92, (2) low risk: 92–98, and (3) no risk: > 98 [22].

### 2.3. Definition of DN

In this research, diabetes was characterized by patients who either self-reported a diabetes diagnosis, were utilizing insulin or oral hypoglycemic agents, exhibited a plasma glycated hemoglobin A1c (HbA1c) level of 6.5% or higher, a fasting blood glucose level of 7.0 mmol/L or above, or a random blood glucose level exceeding 11.1 mmol/L. The diagnosis of DN was assigned to diabetic patients with an estimated glomerular filtration rate (eGFR) below 60 mL/min/1.73m^2^ and/or a UACR of 30 mg/g or more.

The calculation of the eGFR utilizes the CKD-EPI (Chronic Kidney Disease Epidemiology Collaboration) equation, detailed as follows: [23].

For males:

If serum creatinine ≤ 0.9 mg/dL, eGFR = 144 × (serum creatinine/0.9) − 0.411 × 0.993 × age (year).

If serum creatinine > 0.9 mg/dL, eGFR = 144 × (serum creatinine/0.9) − 1.209 × 1.209 × age (year).

For females:

If serum creatinine ≤ 0.7 mg/dL, eGFR = 144 × (serum creatinine/0.7) − 0.329 × 0.329 × age (year).

If serum creatinine > 0.7 mg/dL, eGFR = 144 × (serum creatinine/0.7) − 1.209 × 1.209 × age (year).

### 2.4. Assessment of Covariates

In this study, covariates were meticulously collected from demographic databases, including age, gender, body mass index (BMI), race, education level, marital status, and poverty index. Race was categorized into five groups: Mexican American, other Hispanic, non-Hispanic White, non-Hispanic Black, and other race. Education levels were divided into three categories: below high school, high school graduate, and college graduate or above. Marital status was segmented into three classifications: married or living with a partner; divorced, separated, or widowed; and never married. The poverty income ratio (PIR) was delineated into three ranges: below 1.0, 1.0–3.0, and above 3.0. Comprehensive blood biochemistry tests, including alanine aminotransferase, aspartate aminotransferase, blood urea nitrogen (BUN), total cholesterol, triglyceride, blood uric acid, glycohemoglobin, sodium, and potassium levels, were extracted from laboratory data. Data on hypertension, stroke, cancer, CVD, smoking habits, and alcohol consumption were acquired through questionnaire surveys. Specifically, participants who were informed by a physician of having congestive heart failure, coronary heart disease, and angina/angina pectoris or who had experienced a heart attack were classified as having CVD. The family income to poverty ratio was categorized similarly to the poverty index, with intervals at below 1.0, 1.0–3.0, and 3.0 or higher, where a higher ratio indicates a greater income level relative to the poverty threshold. Regarding smoking status, participants were classified as nonsmokers, former smokers, or current smokers based on their smoking history of having smoked at least 100 cigarettes in their lifetime and their current smoking status.

### 2.5. Ascertainment of Mortality

The principal outcomes of this study include CVD mortality and all-cause mortality. The mortality status of participants was ascertained using the NHANES public-linked mortality files, which facilitate longitudinal tracking of NHANES participants. These files integrate probability matching results between NHANES records and the National Death Index to verify the vital status of each participant. The cause of death is determined according to the International Classification of Diseases, Tenth Revision (ICD-10), with codes I00–I78 specifically designated for deaths related to CVD [24].

The time of event occurrence was calculated from the date of the NHANES examination until either the date of death or the end of the follow-up period on December 31, 2018, whichever occurred first. This methodology ensures a comprehensive evaluation of the mortality outcomes over the study period. Follow-up time was calculated from the date of baseline examination to the date of death or the end of follow-up. The median follow-up time was 2.08 years, and the maximum follow-up duration was 19.58 years.

### 2.6. Statistical Analysis

Data collection, statistical analysis, and interpretation were conducted from October 2023 to February 2024. Given the complex sampling design of NHANES, all analyses in this study incorporated sample weighting, clustering, and stratification. Continuous variables were analyzed using analysis of variance, while categorical variables were evaluated using the chi-square test to compare baseline characteristics across the three GNRI groups. Categorical variables were presented as counts (percentages), and continuous variables were presented as means (standard deviations).

A multivariable Cox proportional hazards model was employed to estimate hazard ratios (HRs) and 95% confidence intervals (CIs) for GNRI, both as a categorical and continuous variable, in relation to CVD mortality and all-cause mortality, with the no risk group serving as the reference. The Schoenfeld residuals test was used to verify the proportional hazards assumption, and no significant deviations from this baseline over time were identified.

All analyses were conducted using survey procedures in R, Version 4.3.1. All hypothesis tests were two-sided, and a *p* value of less than 0.05 was deemed statistically significant. We constructed three models: the first model was a crude model; Model 2 adjusted for age, race, education, marital status, and PIR; Model 3 further adjusted for biochemical and clinical variables including glutamic pyruvic transaminase (U/L), glutamic oxaloacetic transaminase (U/L), BUN (mmol/L), cholesterol (mmol/L), triglycerides (mmol/L), uric acid (mg/dL), creatinine (*μ*mol/L), sodium (mmol/L), potassium (mmol/L), and glycohemoglobin (%), as well as factors such as alcohol consumption, hypertension, CVD, stroke, cancer, and smoking status. To avoid multicollinearity, we first conducted variance inflation factor (VIF) analysis. Variables with a VIF greater than 5 were considered to exhibit multicollinearity and were therefore excluded from the multivariable model. The final set of covariates included in the model was both clinically relevant and statistically independent, ensuring the robustness and interpretability of the model. To explore the nonlinear relationship between continuous GNRI and both CVD mortality and all-cause mortality, we fitted a restricted cubic spline model with four knots placed at the 25th, 50th, 75th, and 100th percentiles. The nonlinearity was tested using the likelihood ratio test.

We conducted subgroup analyses based on age categories (60–75 and ≥ 75 years), gender, race, educational level, marital status, PIR, and the presence of CVD, cancer, hypertension, stroke, as well as alcohol consumption and smoking habits. By calculating the *p* values for the interactions between the GNRI and each stratifying factor, we assessed the significance of these interactions. Furthermore, to test the robustness of our primary findings, we performed several sensitivity analyses. To minimize potential reverse causality bias, participants who died within 2 years of follow-up were excluded. Additionally, to further substantiate the robustness of our main results, we excluded individuals with CVD, cancer, stroke, or hypertension and applied a multivariable Cox proportional hazards model to the remaining dataset. These sensitivity analyses helped us understand the association between GNRI and various health outcomes more accurately.

## 3. Result

### 3.1. Characteristic of Participants

**Figure 1:**
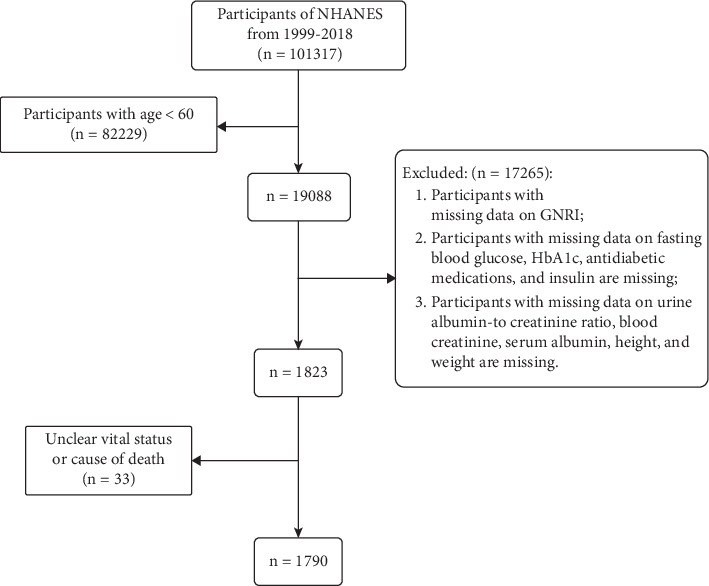
Selection of study participants from the 1999–2018 cycles of the National Health and Nutrition Examination Survey (NHANES).

After applying the exclusion criteria, our analysis included 1790 participants aged 60 and above diagnosed with DN (Figure 1) (average age [SD] 71.24 [7.17] years; males [weighted, 55.0%], females [weighted, 45.0%]). The cohort comprised 395 Mexican American participants (weighted 22.07%), 146 other Hispanic participants (weighted 8.16%), 657 non-Hispanic White participants (weighted 36.70%), 455 non-Hispanic Black participants (weighted 25.42%), and 137 participants of other races or ethnicities (weighted 7.65%). Over a follow-up period totaling 11,724 person-years (average [SE] follow-up time of 6.56 [4.59] years), there were 211 (11.8%) CVD deaths and 914 (51.6%) all-cause mortality cases. The average GNRI value (SD) is reported as follows. According to the GNRI classification, Table 1 details the demographic characteristics of participants in the no risk (*n* = 1299) (72.3%), low risk (*n* = 241) (13.5%), and medium/severe risk (*n* = 250) (14.0%) categories. Compared to the no risk group, the low risk group had a higher proportion of female patients and non-Hispanic Black individuals and lower levels of ALT. Trends of increasing blood creatinine, uric acid, and potassium levels were observed across the groups.

### 3.2. GNRI and All-Cause Mortality

**Figure 2:**
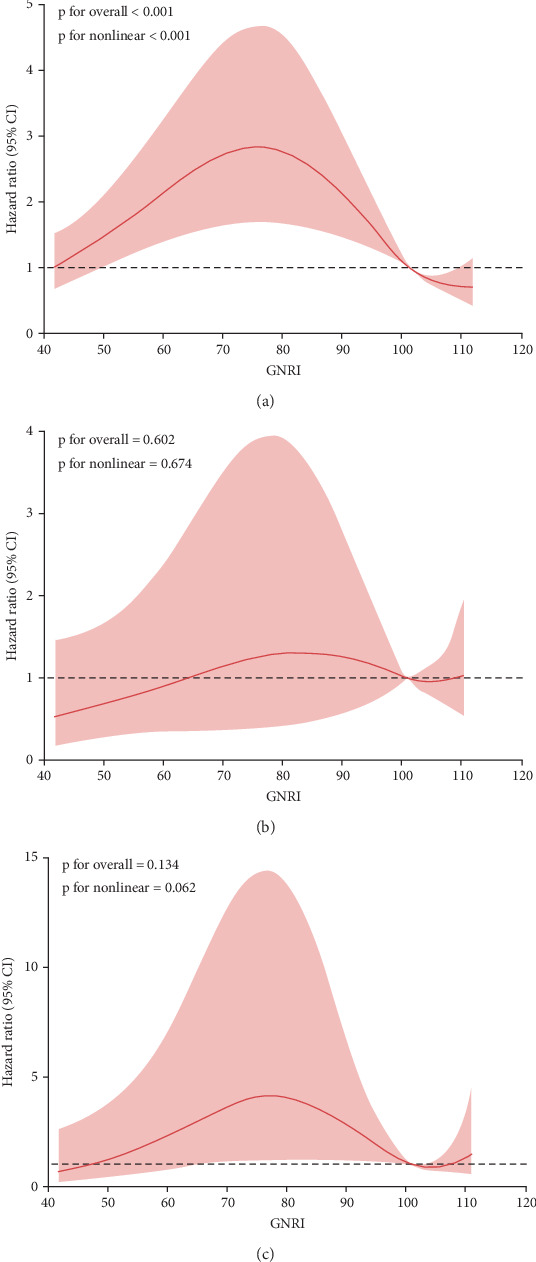
Association between prognostic Geriatric Nutritional Risk Index (GNRI) and (a) all-cause mortality, (b) cardiovascular disease (CVD) mortality, and (c) diabetes mortality in US elderly adults with diabetic nephropathy in the National Health and Nutrition Examination Survey (NHANES) study from 1999 to 2018. Hazard ratios (solid lines) and 95% CIs (shaded areas) were estimated after adjusting for age, sex, race and ethnicity, education level, marital status, poverty income ratio, alcohol, hypertension, cardiovascular disease, stroke, cancer, and smoking.

Among the 1790 DN patients included in the study, 914 (51.6%) experienced all-cause mortality. The distribution of deaths across the no risk, low risk, and M/S risk groups was 620 (34.6%), 137 (7.6%), and 157 (8.8%), respectively. In the multivariable-adjusted Model 1, compared to the no risk group, the M/S risk group had a HR for all-cause mortality of 1.75 (95% CI: 1.47–2.09). In Model 2, the HR was 1.66 (95% CI: 1.17–2.34), and in Model 3, it was 2.43 (95% CI: 1.25–5.80). These adjusted models reveal a progressively increasing trend in HR for all-cause mortality across the no risk, low risk, and M/S risk groups, indicating that a decrease in GNRI is associated with a higher risk of all-cause mortality (Table 2). A restricted cubic spline analysis showed a significant nonlinear relationship between GNRI and the rate of all-cause mortality (*p* for nonlinearity < 0.001) (Figure 2).

### 3.3. GNRI and CVD Mortality

Among all deceased patients, 274 (15.3%) died from CVD, with the distribution across the no risk, low risk, and M/S risk groups being 192 (10.7%), 45 (2.5%), and 37 (2.1%), respectively. In the three multivariable-adjusted models, compared to the no risk group, the HR for all-cause mortality in the M/S risk group was 1.22 (95% CI: 0.86–1.74), 0.45 (95% CI: 0.15–1.30), and 0.52 (95% CI: 0.28–5.22), respectively. No significant statistical relationship was found between GNRI and CVD mortality in patients with DN (Table 2). A restricted cubic spline analysis indicated a significant nonlinear relationship between GNRI and CVD mortality (*p* for nonlinearity < 0.001) (Figure 2).

### 3.4. GNRI and CVD Mortality

Among all decedents, 112 (6.2%) died from CVD; specifically, 75 (4.2%) were in the no risk group, 20 (1.1%) in the low risk group, and 17 (0.9%) in the M/S risk group. In three multivariable-adjusted models, compared with the no risk group, the HRs for all-cause mortality in the M/S risk group were 1.20 (95% CI: 0.70–2.05), 1.15 (95% CI: 0.65–1.98), and 1.18 (95% CI: 0.60–1.90), respectively. No statistically significant association was observed between GNRI and diabetes mortality among patients with DN (Table 2). A restricted cubic spline analysis indicated a significant nonlinear relationship between GNRI and CVD mortality (*p* for nonlinearity < 0.001) (Figure 2).

### 3.5. Subgroup and Sensitivity Analyses

Subgroup analyses were conducted based on gender, age (> 75 vs. ≤ 75 years), race, education level, marital status, PIR, presence of CVD, cancer, hypertension, stroke, alcohol consumption, and smoking habits to explore interactions between different levels of GNRI risk and all-cause and cardiovascular mortality. The results indicated significant interactions between gender, age, and the presence of cancer with all-cause mortality (*p* < 0.001) (Table 3). For cardiovascular mortality, only age showed a significant interaction (*p* < 0.001) (Table 4). For diabetes mortality, no significant interactions were observed across subgroups (Table S1). Therefore, for all-cause mortality, the primary analysis results were reiterated for subgroups with significant interactions: regardless of age group, both > 75 and ≤ 75 years were associated with all-cause mortality but not with cardiovascular mortality (Table S2 and Figure S1). The presence of cancer did not affect the relationship between GNRI and all-cause mortality (Table S3 and Figure S2), aligning with the main conclusions.

However, when analyzing by gender, we found that in male patients, compared to the no risk group, the M/S risk group's HR for all-cause mortality in Model 3 was 2.50 (1.04~5.99). For female patients, the HR was 1.62 (0.83~3.15) (Table S4 and Figure S3).

In sensitivity analyses, patients who died within the first 2 years, those with hypertension, stroke, alcohol, or chronic CVD, were excluded, and the relationship between different GNRI categories and both all-cause and cardiovascular mortality was analyzed. All sensitivity analysis results consistently showed that as GNRI risk increased, so did the rate of all-cause mortality, but no statistical link was found with CVD mortality (Tables S5–S8 and Figures S4–S7), confirming the main findings.

## 4. Discussion

The findings from a comprehensive study utilizing a large national sample in the United States reveal a significant correlation between the GNRI and the risk of all-cause mortality among elderly patients with DN. As GNRI scores decline, the rate of all-cause mortality escalates in this demographic. However, no significant link was established between GNRI and the risk of cardiovascular mortality in these patients. These results persisted across multiple subgroups and sensitivity analyses. Notably, gender-specific analysis demonstrated a more marked association between lower GNRI scores and increased all-cause mortality risk among male elderly patients with DN compared to their female counterparts.

To our knowledge, this is the first study to focus on the relationship between GNRI and all-cause mortality as well as CVD mortality in elderly patients with DN. GNRI, as a measure of nutritional status, has demonstrated predictive value for adverse outcomes across various studies. However, DN patients have kidney-specific pathological changes, and since they are elderly, their overall physical condition is poorer. The relationship between GNRI and these factors is even more worth exploring, as it has significant implications for early guidance and intervention in this patient group. The meta-analysis by Hao et al., encompassing 17,097 patients, substantiates this assertion [25–27]. However, its applicability is limited in certain conditions; for example, Li et al. found no significant correlation between GNRI and overall mortality risk in patients with diffuse large B cell lymphoma [28]. Our study aligns with previous research to a degree. A retrospective study involving 2791 chronic kidney disease patients showed that low GNRI scores are independently associated with overall mortality. Shen et al.'s study also supports this finding, indicating that lower GNRI levels are associated with mortality in older patients with diabetes [22, 29]. The observed independent association between GNRI scores and overall mortality in our study might be attributed to malnutrition, which compromises immune function and enhances the release of inflammatory mediators. These mediators can trigger oxidative stress by promoting free radical production or impairing antioxidant defenses. Consequently, elevated cellular oxidative stress may increase the synthesis and deposition of glomerular matrix proteins, leading to damage and fibrosis in glomerular and tubular epithelial cells, thereby exacerbating renal dysfunction [30–32]. Additionally, malnutrition can cause a decline in muscle mass and functional capabilities in elderly patients, impacting metabolic balance, homeostasis, and immune competence, thus elevating the risk of overall mortality in elderly patients with DN.

In addition, we further examined the relationship between GNRI and diabetes-specific mortality. The results showed that GNRI was not significantly associated with the risk of diabetes-related death, and this finding remained consistent across multiple subgroups and sensitivity analyses. This observation aligns with our main findings—GNRI was significantly associated with all-cause mortality but not with cardiovascular mortality—suggesting that GNRI is more likely to reflect systemic risks related to frailty and malnutrition rather than serve as an independent and stable predictor of diabetes-specific mortality. Clinically, GNRI may be more suitable for overall prognostic stratification and nutritional intervention in elderly patients with DN, while the prevention and management of diabetes-specific death should focus primarily on metabolic control and the management of complications.

However, this study did not identify the GNRI as an independent risk factor for cardiovascular mortality in elderly patients with DN. For diabetic patients, risk factors such as hyperglycemia, hyperlipidemia, and chronic inflammation can promote the progression of atherosclerosis and thrombosis, accelerating the development of CVDs. The independence of GNRI's reduction in relation to these outcomes has also been confirmed in various studies [14, 22, 33]. A meta-analysis by Hung et al. [34] demonstrated that among hemodialysis patients, the risk of cardiovascular mortality increases as GNRI scores decrease. Another study followed 161 nondialysis chronic kidney disease patients over 7 years and found that after adjusting for variables such as gender, hypertension, and diabetes, GNRI independently predicted cardiovascular events [35]. However, these publications, being meta-analyses, are subject to selection biases and did not exclusively involve patients over the age of 60. Elderly DN is a complex condition influenced by multiple factors including sustained hyperglycemia, renal impairment, inflammatory responses, and oxidative stress [36, 37]. As GNRI solely assesses nutritional status, it may not fully capture these complex disease mechanisms after adjusting for multiple covariates. Thus, it may lack independent predictive power for cardiovascular mortality. Factors such as the progression of diabetes, renal impairment, and hyperlipidemia might more directly influence the occurrence and progression of CVDs, thereby diminishing the independence of GNRI in predicting cardiovascular mortality risks in elderly patients with DN [38–40].

Another significant finding of this study is the gender-specific effects observed in subgroup analyses, where the GNRI emerged as an independent risk factor for all-cause mortality in elderly male patients with DN but not in female patients. This disparity may result from physiological differences, lifestyle and behavioral patterns, and sociocultural factors. Previous research indicates that the onset and progression of DN are substantially influenced by gender [41, 42]. A prospective cohort study involving 1470 Type 2 diabetes mellitus (T2DM) patients not on renal replacement therapy (60% male; age, 65 ± 11 years; median follow-up, 5.7 years) identified maleness as an independent factor associated with renal function decline [43]. Testosterone is believed to promote the progression of chronic kidney disease, whereas estrogen is considered to have renal protective effects [44]. Studies have shown that human proximal tubule cells exposed to testosterone undergo rapid phosphorylation by c-Jun amino-terminal kinase (JNK), leading to increased apoptosis of renal proximal tubule cells [45, 46]. Estrogen may mitigate tubulointerstitial fibrosis by inhibiting the synthesis of type I/IV collagen in mesangial cells and promoting the degradation of the extracellular matrix [47], resulting in higher incidence and progression rates of DN in male patients compared to females of the same age group. Men and women have different levels of sex hormones, leading to significant differences in muscle loss. Elderly men typically maintain higher muscle mass and strength throughout their lifetime, while elderly women generally have lower muscle mass and strength. Additionally, changes in muscle mass in women during puberty and menopause are influenced by estrogen levels. The decline in estrogen levels after menopause may exacerbate muscle atrophy. The differences in muscle loss could also be a contributing factor to this outcome. Furthermore, primary human proximal tubular epithelial cells (PTECs) from healthy males exhibit larger volume and mitochondria compared to those from healthy females. When exposed to high glucose, male PTECs exhibit increased mitochondrial respiration, oxidative stress, and apoptotic damage. Metabolomic analysis of blood revealed that male PTECs have increased glucose and glutamine flux through the tricarboxylic acid (TCA) cycle, whereas female PTECs show an increase in pyruvate content. Serum pyruvate levels correlate positively with eGFRs, with male gender and diabetes associated with increased plasma TCA cycle metabolites, which are related to all-cause mortality [48].

Considering the potential biases caused by patients who died within 2 years and those with comorbid conditions such as hypertension, stroke, and chronic CVDs, our further analysis—after excluding these confounding factors—still indicates a correlation between lower GNRI scores and increased risk of all-cause mortality. However, no significant association was found between GNRI and cardiovascular mortality among elderly patients with DN. This suggests that our findings have broad clinical applicability and are not limited by specific patient characteristics, thereby enhancing the reliability and validity of our conclusions.

## 5. Strengths and Limitations

This study boasts several strengths, notably its use of a representative national sample, enhancing the generalizability of the findings to all elderly patients across the United States. Additionally, the comprehensive and high-quality data collection inherent in NHANES facilitates effective control of potential confounding factors, including diverse demographic, socioeconomic, and health-related variables.

However, the study also confronts several limitations. Primarily, the reliance on single-time measurements for serum albumin and estimated eGFR, based on serum creatinine, might not accurately reflect the longitudinal health status or changes in patients. Due to data limitations, changes in GNRI during follow-up were not assessed, which may affect the in-depth evaluation of its long-term prognostic value. Second, data on comorbidities such as hypertension and CVDs were derived from questionnaire surveys, potentially introducing retrospective bias. Being an observational study, it is also unable to establish causal relationships between GNRI and DN. Since frailty cannot be measured, it is impossible to determine the impact of this factor on the differences in mortality between elderly men and women in the subgroup analysis.

## 6. Conclusion

Our study, which analyzed a representative cohort of elderly DN patients in the United States, revealed that lower GNRI scores correlate with a higher risk of all-cause mortality, a relationship that was particularly pronounced among male patients. While a nonlinear relationship was observed between GNRI and all-cause mortality, no significant association was found between GNRI and cardiovascular mortality. These findings suggest that maintaining higher GNRI levels may potentially reduce the risk of all-cause mortality in this patient population.

## Figures and Tables

**Table 1:** Baseline characteristics of older adults with ND in NHANES 1998–2018^a^.

**Variables**	**Total**	**No risk**	**Low risk**	**M/S risk**	**p** ** value ** ^**b**^
		**(** **G** **N** **R** **I** > 98**)**	**(**92 < **GNRI** < 98**)**	**(** **G** **N** **R** **I** < 92**)**	
	**(** **n** = 1790**)**	**(** **n** = 1299**)**	**(** **n** = 241**)**	**(** **n** = 250**)**	
Age, mean (SD), year	71.24 (7.17)	71.09 (7.08)	71.91 (7.37)	71.42 (7.44)	0.238
Gender, *n* (%)					0.002
Male	1002 (55.98)	752 (57.89)	110 (45.64)	140 (56.00)	
Female	788 (44.02)	547 (42.11)	131 (54.36)	110 (44.00)	
BMI					0.013
Male	30.08 (5.75)	30.05 (5.54)	30.50 (6.18)	29.87 (6.85)	
Female	31.59 (7.03)	31.19 (6.85)	33.05 (7.76)	32.03 (6.83)	
Race, *n* (%)					0.002
Mexican American	395 (22.07)	288 (22.17)	53 (21.99)	54 (21.60)	
Other Hispanic	146 (8.16)	109 (8.39)	12 (4.98)	25 (10.00)	
Non-Hispanic White	657 (36.70)	498 (38.34)	80 (33.20)	79 (31.60)	
Non-Hispanic Black	455 (25.42)	296 (22.79)	83 (34.44)	76 (30.40)	
Other race	137 (7.65)	108 (8.31)	13 (5.39)	16 (6.40)	
Education, *n* (%)					0.461
< High school	835 (46.83)	595 (46.02)	113 (46.89)	127 (51.00)	
High school	387 (21.70)	293 (22.66)	47 (19.50)	47 (18.88)	
Some college or above	561 (31.46)	405 (31.32)	81 (33.61)	75 (30.12)	
Marital status, *n* (%)					0.055
Married or living with partner	967 (54.39)	722 (55.97)	119 (49.58)	126 (50.81)	
Divorced, separated, or widowed	717 (40.33)	504 (39.07)	111 (46.25)	102 (41.13)	
Never married	94 (5.29)	64 (4.96)	10 (4.17)	20 (8.06)	
PIR, *n* (%)					0.143
< 1.0	408 (25.87)	289 (24.98)	51 (23.83)	68 (33.01)	
1.0–3.0	813 (51.55)	599 (51.77)	116 (54.21)	98 (47.57)	
> 3.0	356 (22.57)	269 (23.25)	47 (21.96)	40 (19.42)	
Hypertension, *n* (%)					0.759
No	441 (24.64)	326 (25.10)	57 (23.65)	58 (23.20)	
Yes	1349 (75.36)	973 (74.90)	184 (76.35)	192 (76.80)	
Cardiovascular disease, *n* (%)					0.051
No	1176 (65.70)	873 (67.21)	156 (64.73)	161 (64.40)	
Yes	614 (34.30)	426 (32.79)	85 (35.27)	89 (35.60)	
Stroke, *n* (%)					0.092
No	1528 (85.36)	1122 (86.37)	203 (84.23)	203 (81.20)	
Yes	262 (14.64)	177 (13.63)	38 (15.77)	47 (18.80)	
Cancer, *n* (%)					0.259
No	1455 (81.28)	1068 (82.22)	190 (78.84)	197 (78.80)	
Yes	335 (18.72)	231 (17.78)	51 (21.16)	53 (21.20)	
Alcohol, *n* (%)					0.592
No	310 (52.10)	226 (51.83)	37 (48.68)	47 (56.63)	
Yes	285 (47.90)	210 (48.17)	39 (51.32)	36 (43.37)	
Smoking, *n* (%)					0.985
Never smoker	821 (45.87)	595 (45.80)	111 (46.06)	115 (46.00)	
Ever smoker	243 (13.58)	173 (13.32)	34 (14.11)	36 (14.40)	
Current smoker	726 (40.56)	531 (40.88)	96 (39.83)	99 (39.60)	
Blood biochemistry, mean (SD)					
ALT (U/L)	23.17 (24.29)	23.84 (26.34)	19.54 (11.81)	22.93 (18.06)	0.047
AST (U/L)	25.01 (15.97)	25.23 (16.32)	22.63 (10.13)	26.95 (19.99)	0.026
BUN (mmol L)	7.46 (4.01)	7.14 (3.78)	8.24 (4.03)	9.29 (5.32)	< 0.001
TC (mmol/L)	4.73 (1.25)	4.74 (1.23)	4.69 (1.25)	4.73 (1.48)	0.846
TG (mmol/L)	2.16 (1.73)	2.17 (1.62)	2.03 (1.71)	2.24 (2.57)	0.420
Uric acid (mg/dL)	6.04 (1.70)	5.95 (1.67)	6.25 (1.81)	6.47 (1.71)	< 0.001
Sodium (mmol/L)	139.04 (3.10)	139.05 (2.96)	139.11 (3.48)	138.79 (3.71)	0.611
Potassium (mmol/L)	4.19 (0.44)	4.17 (0.44)	4.21 (0.46)	4.30 (0.44)	0.003
Glycohemoglobin (%)	7.56 (1.76)	7.52 (1.69)	7.67 (1.99)	7.68 (1.91)	0.307
GNRI, mean (SD)	96.24 (17.79)	103.88 (3.82)	95.35 (1.52)	57.35 (18.89)	< 0.001
Follow-up (year)	6.56 (4.59)	7.01 (4.64)	5.22 (4.10)	5.50 (4.33)	0.142
CVD mortality					
No	640 (70.02)	428 (69.03)	92 (67.15)	120 (76.43)	
Yes	274 (29.98)	192 (30.97)	45 (32.85)	37 (23.57)	
All-cause mortality					< 0.001
No	874 (48.88)	678 (52.23)	103 (42.92)	93 (37.20)	
Yes	914 (51.12)	620 (47.77)	137 (57.08)	157 (62.80)	
DN					
UACR < 30 mg/g	49 (2.75)	4 (1.66)	3 (1.2)	42 (3.24)	< 0.001
30 ≤ UACR ≤ 300 mg/g	1283 (71.88)	154 (63.9)	146 (58.63)	983 (75.91)	
UACR > 300 mg/g	453 (25.38)	83 (34.44)	100 (40.16)	270 (20.85)	

Abbreviations: ALT, alanine aminotransferase; AST, aspartate aminotransferase; BMI, body mass index; BUN, blood urea nitrogen; DN, diabetic nephropathy; GNRI, Geriatric Nutritional Risk Index; NHANES, National Health and Nutrition Examination Survey; PIR, poverty income ratio; TC, total cholesterol; TG, triglyceride.

^a^All estimates accounted for complex survey designs, and all percentages are weighted.

^b^ *p* < 0.05 was considered statistically significant.

**Table 2:** Association of GNRI with all-cause and CVD-related mortality among older adults with DN from the 1998 to 2018 cycles of the National Health and Nutrition Examination Survey.

**Model**	**Hazard ratio (95% CI)**			**p** **^d^ value for trend**
	**No risk (** **G** **N** **R** **I** > 98**)**	**Low risk (**92 < **GNRI** < 98**)**	**M/S risk (** **G** **N** **R** **I** < 92**)**	
All-cause mortality				
Total deaths, no.	620/1299	137/241	157/250	NA
Model 1^a^	1.00 (reference)	1.70 (1.41~2.05)	1.75 (1.47~2.09)	< 0.001
Model 2^b^	1.00 (reference)	1.32 (0.88~1.96)	1.66 (1.17~2.34)	0.004
Model 3^c^	1.00 (reference)	1.56 (1.19~2.38)	2.43 (1.25~5.80)	0.021
CVD mortality				
Total deaths, no.	129/1299	45/241	37/250	NA
Model 1^a^	1.00 (reference)	1.66 (1.20~2.30)	1.22 (0.86~1.74)	0.269
Model 2^b^	1.00 (reference)	1.09 (0.51~2.34)	0.45 (0.15~1.30)	0.139
Model 3^c^	1.00 (reference)	0.89 (0.43~2.14)	0.52 (0.28~5.22)	0.538
Diabetes mortality				
Total deaths, no.	75/1299	20/241	17/250	NA
Model 1^a^	1.00 (reference)	1.35 (0.80–2.25)	1.20 (0.70–2.05)	0.182
Model 2^b^	1.00 (reference)	1.28 (0.75–2.18)	1.15 (0.65–1.98)	0.225
Model 3^c^	1.00 (reference)	1.30 (0.72–2.25)	1.18 (0.60–1.90)	0.256

Abbreviations: CI, confidence interval; CVD, cardiovascular disease; DN, diabetic nephropathy; GNRI, Geriatric Nutritional Risk Index.

^a^Crude model.

^b^Adjusted for age, gender, race, education, marital status, and poverty income ratio.

^c^Adjusted for age, gender, race, education, marital status, poverty income ratio, alanine aminotransferase (U/L), aspartate aminotransferase (U/L), BUN (mmol L), cholesterol (mmol/L), triglycerides (mmol/L), uric acid (mg/dL), creatinine (*μ*mol/L), sodium (mmol/L), potassium (mmol/L), glycohemoglobin (%), white blood cells, lymphocytes, monocytes, C-reactive protein, alcohol, hypercholesterolemia, hypertension, cardiovascular disease, stroke, cancer, and smoking.

^d^ *p* < 0.05 was considered statistically significant.

**Table 3:** Stratified analysis of the association between GNRI and all-cause mortality in patients with older DN in NHANES 1999–2018.

**Variables**	**No risk**	**Low risk (HR, 95% CI)**	**M/S risk (HR, 95% CI)**	**p** ** value (trend)**	**p** ** value (interaction)**
	**(** **G** **N** **R** **I** > 98**)**	**(GNRI: 92–98)**	**(** **G** **N** **R** **I** < 92**)**		
Age, years					*p* < 0.001
≤ 75	Reference	0.516 (0.410–0.649)	0.877 (0.643–1.196)	*p* < 0.001	
> 75	Reference	0.674 (0.513–0.887)	1.081 (0.767–1.524)	*p* < 0.001	
Sex					*p* < 0.001
Male	Reference	0.485 (0.379–0.621)	0.802 (0.568–1.132)	*p* < 0.001	
Female	Reference	0.641 (0.470–0.875)	1.105 (0.759–1.608)	*p* < 0.001	
Race					*p* = 0.697
Mexican American	Reference	0.408 (0.279–0.598)	0.916 (0.564–1.489)	*p* < 0.001	
Other Hispanic	Reference	0.715 (0.351–1.454)	0.578 (0.157–2.216)	*p* = 0.581	
Non-Hispanic White	Reference	0.572 (0.430–0.760)	1.067 (0.737–1.544)	*p* < 0.001	
Non-Hispanic Black	Reference	0.576 (0.413–0.804)	1.015 (0.672–1.535)	*p* < 0.001	
Other race	Reference	0.379 (0.169–0.849)	0.334 (0.070–1.581)	*p* = 0.055	
Educational level					*p* = 0.944
< High school	Reference	0.630 (0.483–0.821)	0.936 (0.660–1.326)	*p* < 0.001	
High school	Reference	0.546 (0.351–0.848)	0.987 (0.564–1.729)	*p* = 0.002	
Some college or above	Reference	0.493 (0.339–0.716)	0.827 (0.513–1.333)	*p* < 0.001	
Marital status					*p* = 0.751
Married or living with partner	Reference	0.473 (0.365–0.614)	0.891 (0.626–1.270)	*p* < 0.001	
Divorced, separated, or widowed	Reference	0.711 (0.525–0.963)	0.982 (0.675–1.428)	*p* = 0.016	
Never married	Reference	0.735 (0.325–1.660)	0.540 (0.140–2.079)	*p* = 0.628	
PIR					*p* = 0.410
< 1.0	Reference	0.611 (0.424–0.879)	1.024 (0.624–1.681)	*p* = 0.005	
1.0–3.0	Reference	0.553 (0.426–0.719)	0.747 (0.534–1.045)	*p* < 0.001	
> 3.0	Reference	0.575 (0.358–0.926)	1.454 (0.798–2.650)	*p* < 0.001	
CVD					*p* = 0.084
Yes	Reference	0.577 (0.421–0.790)	0.967 (0.655–1.426)	*p* < 0.001	
No	Reference	0.602 (0.471–0.769)	0.893 (0.643–1.241)	*p* < 0.001	
Cancer					*p* = 0.027
Yes	Reference	0.681 (0.453–1.024)	1.012 (0.611–1.679)	*p* = 0.052	
No	Reference	0.552 (0.443–0.688)	0.855 (0.638–1.144)	*p* < 0.001	
Hypertension					*p* = 0.195
Yes	Reference	0.592 (0.475–0.736)	0.957 (0.718–1.275)	*p* < 0.001	
No	Reference	0.553 (0.371–0.824)	0.779 (0.481–1.327)	*p* = 0.007	
Stroke					*p* = 0.732
Yes	Reference	0.513 (0.328–0.801)	0.567 (0.312–1.031)	*p* < 0.001	
No	Reference	0.584 (0.475–0.724)	0.992 (0.752–1.307)	*p* = 0.013	
Alcohol					*p* = 0.864
Yes	Reference	0.694 (0.408–1.180)	1.567 (0.845–2.906)	*p* < 0.001	
No	Reference	0.588 (0.389–0.890)	0.971 (0.555–1.700)	*p* = 0.012	
Smoking					*p* = 0.369
Never smoker	Reference	0.752 (0.551–1.026)	1.519 (1.022–2.258)	*p* < 0.001	
Ever smoker	Reference	0.451 (0.276–0.738)	0.669 (0.358–1.252)	*p* = 0.005	
Current smoker	Reference	0.499 (0.376–0.662)	0.606 (0.4125–0.892)	*p* < 0.001	
DN					
UACR < 30 mg/g	Reference	1.909 (0.633–5.757)	3.818 (2.298–6.344)	*p* < 0.001	*p* = 0.205
30 ≤ UACR ≤ 300 mg/g	Reference	1.168 (0.995–1.371)	1.342 (1.164–1.548)	*p* < 0.001	
UACR > 300 mg/g	Reference	1.135 (0.931–1.384)	1.142 (0.949–1.373)	*p* = 0.160	

Abbreviations: CVD, cardiovascular disease; DN, diabetic nephropathy; GNRI, Geriatric Nutritional Risk Index; PIR, poverty income ratio.

**Table 4:** Stratified analysis of the association between GNRI and CVD mortality in patients with older DN in NHANES 1999–2018.

**Variables**	**No risk**	**Low risk (HR, 95% CI)**	**M/S risk (HR, 95% CI)**	**p** ** value (trend)**	**p** ** value (interaction)**
	**(** **G** **N** **R** **I** > 98**)**	**(**92 < **GNRI** < 98**)**	**(** **G** **N** **R** **I** < 92**)**		
Age, years					*p* < 0.01
≤ 75	Reference	0.791 (0.457–1.370)	1.339 (0.678–2.643)	*p* = 0.105	
> 75	Reference	0.694 (0.408–1.181)	1.038 (0.537–2.007)	*p* = 0.170	
Sex					*p* = 0.323
Male	Reference	0.512 (0.335–0.783)	1.299 (0.761–2.217)	*p* < 0.01	
Female	Reference	1.583 (0.794–3.158)	1.878 (0.841–4.192)	*p* = 0.301	
Race					*p* = 0.165
Mexican American	Reference	0.442 (0.206–0.948)	0.855 (0.316–2.318)	*p* = 0.064	
Other Hispanic	Reference	0.914 (0.197–4.234)	0.704 (0.058–8.596)	*p* = 0.962	
Non-Hispanic White	Reference	0.721 (0.408–1.274)	1.619 (0.811–3.229)	*p* = 0.005	
Non-Hispanic Black	Reference	1.247 (0.518–3.002)	1.532 (0.548–4.278)	*p* = 0.714	
Other race	Reference	1.131 (0.104–2.275)	1.648 (0.702–4.933)	*p* = 0.995	
Educational level					*p* = 0.298
< High school	Reference	0.845 (0.494–1.446)	0.956 (0.466–1.962)	*p* = 0.783	
High school	Reference	0.578 (0.257–1.301)	1.284 (0.472–3.491)	*p* = 0.071	
Some college or above	Reference	0.754 (0.357–1.591)	1.622 (0.703–3.740)	*p* = 0.047	
Marital status					*p* = 0.095
Married or living with a partner	Reference	0.550 (0.333–0.906)	1.088 (0.570–2.079)	*p* = 0.005	
Divorced, separated, or widowed	Reference	1.431 (0.686–2.986)	2.055 (0.897–4.706)	*p* = 0.191	
Never married	Reference	0.487 (0.166–1.428)	0.320 (0.037–2.775)	*p* = 0.346	
PIR					*p* = 0.617
< 1.0	Reference	0.727 (0.365–1.445)	0.892 (0.326–2.443)	*p* = 0.632	
1.0–3.0	Reference	0.779 (0.458–1.325)	1.146 (0.603–2.179)	*p* = 0.209	
> 3.0	Reference	0.698 (0.266–1.831)	2.495 (0.826–7.533)	*p* = 0.005	
CVD					*p* = 0.079
Yes	Reference	0.784 (0.451–1.363)	1.372 (0.718–2.622)	*p* = 0.052	
No	Reference	0.652 (0.386–1.102)	0.922 (0.454–1.874)	*p* = 0.179	
Cancer					*p* = 0.056
Yes	Reference	0.745 (0.303–1.828)	1.145 (0.393–3.339)	*p* = 0.530	
No	Reference	0.732 (0.481–1.113)	1.264 (0.747–2.139)	*p* = 0.016	
Hypertension					*p* = 0.319
Yes	Reference	0.845 (0.531–1.346)	1.194 (0.663–2.149)	*p* = 0.275	
No	Reference	0.459 (0.235–0.894)	1.010 (0.456–2.235)	*p* = 0.009	
Stroke					*p* = 0.125
Yes	Reference	0.560 (0.250–1.256)	0.391 (0.116–1.319)	*p* = 0.244	
No	Reference	0.755 (0.488–1.167)	1.442 (0.853–2.437)	*p* = 0.003	
Alcohol					*p* = 0.150
Yes	Reference	1.098 (0.335–3.591)	1.376 (0.325–5.828)	*p* = 0.880	
No	Reference	1.378 (0.485–3.916)	2.186 (0.624–7.656)	*p* = 0.428	
Smoking					*p* = 0.099
Never smoker	Reference	1.147 (0.607–2.167)	1.810 (0.818–4.004)	*p* = 0.236	
Ever smoker	Reference	0.275 (0.118–0.637)	0.852 (0.335–2.168)	*p* = 0.003	
Current smoker	Reference	0.713 (0.386–1.318)	0.948 (0.436–2.060)	*p* = 0.405	
DN					
UACR < 30 mg/g	Reference	2.100 (0.686–6.432)	NA	*p* = 0.740	*p* = 0.561
30 ≤ UACR ≤ 300 mg/g	Reference	1.288 (1.044–1.589)	0.900 (0.686–1.180)	*p* = 0.896	
UACR > 300 mg/g	Reference	1.117 (0.826–1.511)	1.282 (0.988–1.663)	*p* = 0.063	

Abbreviations: CVD, cardiovascular disease; DN, diabetic nephropathy; GNRI, Geriatric Nutritional Risk Index; PIR, poverty income ratio.

## Data Availability

All data for this manuscript can be found at https://wwwn.cdc.gov/nchs/nhanes/Default.aspx.

## Supporting Information

Supporting Information Additional supporting information can be found online in the Supporting Information section. The supporting information provides stratified and sensitivity analyses of the association between GNRI and mortality risk in elderly patients with diabetic nephropathy. Tables S1–S8 present subgroup risk estimates, and Figures S1–S7 illustrate the corresponding associations.
