# Supplementary material for: Association of Geriatric Nutritional Risk Index With Cardiovascular and All-Cause Mortality Among US Elderly Adults With Diabetic Nephropathy
Source: J Diabetes Res. 2025 Oct 23;2025:2746706. doi: 10.1155/jdr/2746706 (PMC12575020; doi:10.1155/jdr/2746706)
Supplement: Supporting Information — Additional supporting information can be found online in the Supporting Information section. The supporting information provides stratified and sensitivity analyses of the association between GNRI and mortality risk in elderly patients with diabetic nephropathy. Tables S1–S8 present subgroup risk estimates, and Figures S1–S7 illustrate the corresponding associations. [file 2746706.f1.docx]

**Supplement Material:**

Table S1. Stratified Analysis of the Association Between GNRI and diabetes Mortality in Patients with Older DN in NHANES 1999–2018

Table S2. Adjusted Hazard Ratios and 95% CIs of GNRI With Risk of All-Cause Mortality and Cardiovascular Disease Mortality Risk for Older Adults with DN Stratified by Age.

Table S3. Adjusted Hazard Ratios and 95% CIs of GNRI With Risk of All-Cause Mortality for Older Adults with DN Stratified by Gender.

Table S4. Adjusted Hazard Ratios and 95% CIs of GNRI With Risk of All-Cause Mortality for Older Adults with DN Stratified by Cancer.

Table S5. Adjusted Hazard Ratios and 95% CIs of GNRI With Risk of All-Cause Mortality and CVD Mortality for Older Adults with DN Stratified After Excluding Death Within the First 2 years of Follow-up.

Table S6. Adjusted Hazard Ratios and 95% CIs of GNRI With Risk of All-Cause Mortality and CVD Mortality for Older Adults with DN Stratified After Excluding Participants with Hypertension.

Table S7. Adjusted Hazard Ratios and 95% CIs of GNRI With Risk of All-Cause Mortality and CVD Mortality for Older Adults with DN Stratified After Excluding Participants with Cardiovascular Disease.

Table S8. Adjusted Hazard Ratios and 95% CIs of GNRI With Risk of All-Cause Mortality and CVD Mortality for Older Adults with DN Stratified After Excluding Participants with Stroke.

Figure S1. Association between prognostic Geriatric Nutritional Risk Index (GNRI)and all-cause mortality and cardiovascular disease (CVD) mortality in US Elderly Adults with Diabetic Nephropathy Stratified by Age.

Figure S2. Association between prognostic Geriatric Nutritional Risk Index (GNRI)and all-cause mortality and cardiovascular disease (CVD) mortality in US Elderly Adults with Diabetic Nephropathy Stratified Stratified by Gender.

Figure S3. Association between prognostic Geriatric Nutritional Risk Index (GNRI)and all-cause mortality and cardiovascular disease (CVD) mortality in US Elderly Adults with Diabetic Nephropathy Stratified by Cancer.

Figure S4. Association between prognostic Geriatric Nutritional Risk Index (GNRI)and all-cause mortality (A) and cardiovascular disease (CVD) mortality (B) in US Elderly Adults with Diabetic Nephropathy Stratified After Excluding Death Within the First 2 years of Follow-up.

Figure S5. Association between prognostic Geriatric Nutritional Risk Index (GNRI)and all-cause mortality (A) and cardiovascular disease (CVD) mortality (B) in US Elderly Adults with Diabetic Nephropathy Stratified After Excluding Participants with Hypertension.

Figure S6. Association between prognostic Geriatric Nutritional Risk Index (GNRI)and all-cause mortality in US Elderly Adults with Diabetic Nephropathy Stratified After Excluding Participants with Cardiovascular Disease.

Figure S7. Association between prognostic Geriatric Nutritional Risk Index (GNRI)and all-cause mortality (A) and cardiovascular disease (CVD) mortality (B) in US Elderly Adults with Diabetic Nephropathy Stratified After Excluding Participants with Stroke.

Table S1. Stratified Analysis of the Association Between GNRI and diabetes Mortality in Patients with Older DN in NHANES 1999–2018

| Variables | No risk | Low risk | M/S risk | P value（trend） | P value (interaction) |
| --- | --- | --- | --- | --- | --- |
|  | (GNRI＞98) | (92＜GNRI＜98) | (GNRI＜92) |  |  |
| Age, years |  |  |  |  | P=.158 |
| ≤75 | Reference | 1.938 (0.964-3.896) | 1.906 (0.947-3.836) | P=0.001 |  |
| ＞75 | Reference | 1.721 (0.733-4.042) | 0.460 (0.107-1.970) | P=0.463 |  |
| Sex |  |  |  |  | P=.024 |
| Male | Reference | 1.333 (0.520-3.417) | 2.263 (1.112-4.607) | P=0.001 |  |
| Female | Reference | 2.216 (1.115-4.407) | 0.397 (0.094-1.676) | P=0.821 |  |
| Race |  |  |  |  | P=.646 |
| Mexican American | Reference | 2.107 (0.694-6.400) | 3.150 (1.215-8.166) | P=0.019 |  |
| Other Hispanic | Reference | 2.112 (0.233-19.152) | 1.269 (0.141-11.402) | P=0.453 |  |
| Non-Hispanic White | Reference | 1.631 (0.626-4.249) | 0.641 (0.152-2.703) | P=0.963 |  |
| Non-Hispanic Black | Reference | 1.617 (0.621-4.212) | 0.611 (0.139-2.683) | P=0.258 |  |
| Other Race | Reference | 5.095 (0.458-56.716) | 2.847 (0.223-36.271) | P=0.218 |  |
| Educational level |  |  |  |  | P=.359 |
| <High school | Reference | 1.769 (0.815-3.840) | 1.355 (0.596-3.080) | P=0.068 |  |
| High school | Reference | 0.517 (0.067-3.969) | 1.839 (0.527-6.417) | P=0.388 |  |
| Some college or above | Reference | 3.120 (1.309-7.435) | 0.890 (0.204-3.889) | P=0.219 |  |
| Marital status |  |  |  |  | P=.159 |
| Married or living with a partner | Reference | 2.193 (1.007-4.772) | 1.947 (0.895-4.235) | P=0.010 |  |
| Divorced, separated, or widowed | Reference | 1.253 (0.544-2.888) | 0.590 (0.179-1.944) | P=0.895 |  |
| Never married | Reference | 6.981 (0.429-113.517) | 0.000 (0.000-inf) | P=0.206 |  |
| PIR |  |  |  |  | P=.635 |
| <1.0 | Reference | 2.402 (0.854-6.753) | 1.888 (0.676-5.269) | P=0.101 |  |
| 1.0-3.0 | Reference | 1.700 (0.893-3.237) | 1.051 (0.474-2.333) | P=0.084 |  |
| >3.0 | Reference | 2.402 (0.854-6.753) | 1.888 (0.676-5.269) | P=0.624 |  |
| CVD |  |  |  |  | P =.295 |
| Yes | Reference | 1.239 (0.503-3.056) | 1.646 (0.701-3.863) | P=0.077 |  |
| No | Reference | 2.300 (1.165-4.542) | 0.936 (0.366-2.393) | P=0.151 |  |
| Cancer |  |  |  |  | P=.053 |
| Yes | Reference | 2.545 (0.941-6.878) | 0.000 (0.000-inf) | P=0.351 |  |
| No | Reference | 1.594 (0.826-3.075) | 1.641 (0.872-3.090) | P=0.005 |  |
| Hypertension |  |  |  |  | P=.316 |
| Yes | Reference | 2.292 (1.263-4.160) | 1.227 (0.597-2.523) | P=0.014 |  |
| No | Reference | 0.789 (0.181-3.437) | 1.466 (0.430-4.995) | P=0.740 |  |
| Stroke |  |  |  |  | P=.137 |
| Yes | Reference | 0.409 (0.054-3.114) | 1.101 (0.309-3.920) | P=0.875 |  |
| No | Reference | 2.350 (1.324-4.174) | 1.306 (0.638-2.672) | P=0.015 |  |
| Alcohol |  |  |  |  | P=.268 |
| Yes | Reference | 4.754 (1.862-12.140) | 1.262 (0.285-5.582) | P=0.080 |  |
| No | Reference | 1.957 (0.685-5.594) | 1.216 (0.413-3.586) | P=0.491 |  |
| Smoking |  |  |  |  | P=.661 |
| Never smoker | Reference | 2.220 (1.001-4.924) | 0.829 (0.289-2.376) | P=0.267 |  |
| Ever smoker | Reference | 1.711 (0.336-8.715) | 0.952 (0.111-8.136) | P=0.631 |  |
| Current smoker | Reference | 1.639 (0.710-3.784) | 2.012 (0.874-4.630) | P=0.036 |  |

Abbreviations: DN: diabetic nephropathy; GNRI, Geriatric Nutrition Risk Index; PIR: poverty income ratio; CVD: cardiovascular disease.

Table S2. Adjusted Hazard Ratios and 95% CIs of GNRI With Risk of All-Cause Mortality and Cardiovascular Disease Mortality Risk for Older Adults with DN Stratified by Age.

| Age, years | Model | Hazard ratio (95% CI) | | | P value for trend ^d^ |
| --- | --- | --- | --- | --- | --- |
|  |  | No risk (GNRI＞98) | Low risk (92＜GNRI＜98) | No risk (GNRI＞98) |  |
| ≤75 | All-cause mortality |  |  |  |  |
|  | Total deaths, No. | 348/1184 | 70/1184 | 93/1184 | NA |
|  | Model 1^a^ | 1.00 (Reference) | 1.70 (1.31 ~ 2.20) | 1.94 (1.54 ~ 2.44) | <.001 |
|  | Model 2^b^ | 1.00 (Reference) | 2.33 (1.46 ~ 3.71) | 1.65 (1.01 ~ 2.69) | .046 |
|  | Model 3^c^ | 1.00 (Reference) | 1.96 (1.19 ~ 3.21) | 2.19 (1.61 ~ 3.17) | .028 |
|  | CVD mortality |  |  |  |  |
|  | Total deaths, No. | 103/1184 | 22/1184 | 18/1184 | NA |
|  | Model 1^a^ | 1.00 (Reference) | 1.58 (0.99 ~ 2.52) | 1.14 (0.69 ~ 1.90) | .604 |
|  | Model 2^b^ | 1.00 (Reference) | 0.49 (0.11 ~ 2.19) | 0.45 (0.10 ~ 2.00) | .296 |
|  | Model 3^c^ | 1.00 (Reference) | 0.40 (0.08 ~ 1.91) | 0.56(0.12 ~ 2.43) | .249 |
| ＞75 | All-cause mortality |  |  |  |  |
|  | Total deaths, No. | 272/606 | 67/606 | 64/606 | NA |
|  | Model 1^a^ | 1.00 (Reference) | 1.60 (1.23 ~ 2.10) | 1.48 (1.13 ~ 1.95) | .005 |
|  | Model 2^b^ | 1.00 (Reference) | 1.54 (1.17 ~ 2.43) | 1.69 (1.52 ~ 2.03) | .006 |
|  | Model 3^c^ | 1.00 (Reference) | 2.09 (1.23 ~ 3.56) | 2.67 (1.36 ~ 5.26) | .004 |
|  | CVD mortality |  |  |  |  |
|  | Total deaths, No. | 89/606 | 23/606 | 19/606 | NA |
|  | Moder 1^a^ | 1.00 (Reference) | 1.82 (1.14 ~ 2.90) | 1.42 (0.86 ~ 2.35) | .169 |
|  | Moder 2^b^ | 1.00 (Reference) | 2.49 (1.06 ~ 5.85) | 0.93 (0.32 ~ 2.66) | .888 |
|  | Moder 3^c^ | 1.00 (Reference) | 1.46 (0.50 ~ 4.27) | 3.32 (0.81 ~ 13.66) | .096 |

Abbreviations: GNRI, Geriatric Nutrition Risk Index; CVD, cardiovascular disease; DN: diabetic nephropathy; CI: Confidence Interval

a: Crude model

b: Adjusted for gender, Race, Education, Marital status, poverty income ratio

c: Adjust for Age, gender, Race, Education, Marital status, poverty income ratio, alanine aminotransferase (U/L), aspartate aminotransferase (U/L), BUN (mmol L), Cholesterol (mmol/L), Triglycerides (mmol/L), Uric acid (mg/dL), Creatinine (umol/L), Sodium (mmol/L), Potassium (mmol/L), Glycohemoglobin (%), White blood cells, lymphocytes, monocytes and C-reactive protein, alcohol, Hypercholesterolemia, hypertension, cardiovascular disease, stroke, Cancer, Smoking;

d P < .05 was considered statistically significant.

Table S3. Adjusted Hazard Ratios and 95% CIs of GNRI With Risk of All-Cause Mortality for older adults with DN Stratified by Gender.

| Gender | Model | Hazard ratio (95% CI) | | | P value for trend ^d^ |
| --- | --- | --- | --- | --- | --- |
|  |  | No risk (GNRI＞98) | Low risk (92＜GNRI＜98) | No risk (GNRI＞98) |  |
| Male | All-cause mortality |  |  |  |  |
|  | Total deaths, No. | 371/1002 | 64/1002 | 96/1002 | NA |
|  | Model 1^a^ | 1.00 (Reference) | 1.84 (1.41 ~ 2.40) | 2.07 (1.66 ~ 2.60) | <.001 |
|  | Model 2^b^ | 1.00 (Reference) | 8.02 (3.94 ~ 16.31) | 2.10 (1.10 ~ 4.02) | <.001 |
|  | Model 3^c^ | 1.00 (Reference) | 9.24 (4.09 ~ 20.89) | 2.50 (1.04 ~ 5.99) | <.001 |
| Female | All-cause mortality |  |  |  |  |
|  | Total deaths, No. | 249/788 | 73/788 | 61/788 | NA |
|  | Model 1^a^ | 1.00 (Reference) | 1.66 (1.28 ~ 2.16) | 1.43 (1.08 ~ 1.89) | .013 |
|  | Model 2^b^ | 1.00 (Reference) | 1.60 (1.08 ~ 2.37) | 1.36 (0.91 ~ 2.04) | .135 |
|  | Model 3^c^ | 1.00 (Reference) | 1.46 (0.97 ~ 2.20) | 1.62 (0.83 ~ 3.15) | .157 |

Abbreviations: GNRI, Geriatric Nutrition Risk Index; CVD, cardiovascular disease; DN: diabetic nephropathy; CI: Confidence Interval

a: Crude model;

b: Adjusted for Age, Race, Education, Marital status, poverty income ratio;

c: Adjust for Age, gender, Race, Education, Marital status, poverty income ratio, alanine aminotransferase (U/L), aspartate aminotransferase (U/L), BUN (mmol L), Cholesterol (mmol/L), Triglycerides (mmol/L), Uric acid (mg/dL), Creatinine (umol/L), Sodium (mmol/L), Potassium (mmol/L), Glycohemoglobin (%), White blood cells, lymphocytes, monocytes and C-reactive protein, alcohol, Hypercholesterolemia, hypertension, cardiovascular disease, stroke, Cancer, Smoking;

d P < .05 was considered statistically significant.

Table S4. Adjusted Hazard Ratios and 95% CIs of GNRI With Risk of All-Cause Mortality for older adults with DN Stratified by Cancer.

| Cancer | Model | Hazard ratio (95% CI) | | | P value for trend |
| --- | --- | --- | --- | --- | --- |
|  |  | No risk (GNRI＞98) | Low risk (92＜GNRI＜98) | No risk (GNRI＞98) |  |
| Yes | All-cause mortality |  |  |  |  |
|  | Total deaths, No. |  |  |  | NA |
|  | Model 1^a^ | 1.00 (Reference) | 1.63 (1.12 ~ 2.39) | 1.75 (1.20 ~ 2.53) | .003 |
|  | Model 2^b^ | 1.00 (Reference) | 2.54 (1.25 ~ 5.16) | 1.59 (1.18 ~ 3.17) | .010 |
|  | Model 3^c^ | 1.00 (Reference) | 2.42 (1.29 ~ 5.92) | 7.05 (1.23 ~ 40.29) | .028 |
| No | All-cause mortality |  |  |  |  |
|  | Total deaths, No. |  |  |  | NA |
|  | Model 1^a^ | 1.00 (Reference) | 1.71 (1.38 ~ 2.11) | 1.74 (1.43 ~ 2.13) | <.001 |
|  | Model 2^b^ | 1.00 (Reference) | 1.89 (1.27 ~ 2.81) | 1.50 (1.01 ~ 2.22) | .045 |
|  | Model 3^c^ | 1.00 (Reference) | 1.68 (1.03 ~ 2.74) | 2.02 (1.10 ~ 3.70) | .023 |

Abbreviations: GNRI, Geriatric Nutrition Risk Index; CVD, cardiovascular disease; DN: diabetic nephropathy; CI: Confidence Interval

a: Crude model

b: Adjusted for Age, Gender, Race, Education, Marital status, poverty income ratio

c: Adjust for Age, gender, Race, Education, Marital status, poverty income ratio, alanine aminotransferase (U/L), aspartate aminotransferase (U/L), BUN (mmol L), Cholesterol (mmol/L), Triglycerides (mmol/L), Uric acid (mg/dL), Creatinine (umol/L), Sodium (mmol/L), Potassium (mmol/L), Glycohemoglobin (%), White blood cells, lymphocytes, monocytes and C-reactive protein, alcohol, Hypercholesterolemia, hypertension, cardiovascular disease, stroke, Cancer, Smoking;

d P < .05 was considered statistically significant.

Table S5. Adjusted Hazard Ratios and 95% CIs of GNRI With Risk of All-Cause Mortality and CVD Mortality for older adults with DN Stratified After Excluding Death Within the First 2 years of Follow-up.

| Model | Hazard ratio (95% CI) | | | P value for trend ^d^ |
| --- | --- | --- | --- | --- |
|  | No risk (GNRI＞98) | Low risk (92＜GNRI＜98) | No risk (GNRI＞98) |  |
| All-cause mortality |  |  |  |  |
| Total deaths, No. | 541/1525 | 98/1525 | 110/1525 | NA |
| Model 1^a^ | 1.00 (Reference) | 1.50 (1.21 ~ 1.86) | 1.47 (1.20 ~ 1.81) | <.001 |
| Model 2^b^ | 1.00 (Reference) | 1.37 (1.08 ~ 1.75) | 1.39 (1.10 ~ 1.75) | .010 |
| Model 3^c^ | 1.00 (Reference) | 1.16 (1.05 ~ 1.50) | 1.52 (1.10 ~ 2.09) | .005 |
| CVD mortality |  |  |  |  |
| Total deaths, No. | 167/1525 | 32/1525 | 23/1525 | NA |
| Model 1^a^ | 1.00 (Reference) | 1.48 (1.01 ~ 2.17) | 0.96 (0.62 ~ 1.48) | 0.840 |
| Model 2^b^ | 1.00 (Reference) | 1.54 (0.98 ~ 2.41) | 1.01 (0.61 ~ 1.67) | 0.971 |
| Model 3^c^ | 1.00 (Reference) | 1.32 (0.82 ~ 2.15) | 1.30 (0.68 ~ 2.51) | 0.428 |

Abbreviations: GNRI, Geriatric Nutrition Risk Index; CVD, cardiovascular disease; DN: diabetic nephropathy; CI: Confidence Interval

a: Crude model;

b: Adjusted for Age, Gender, Race, Education, Marital status, poverty income ratio;

c: Adjust for Age, gender, Race, Education, Marital status, poverty income ratio, alanine aminotransferase (U/L), aspartate aminotransferase (U/L), BUN (mmol L), Cholesterol (mmol/L), Triglycerides (mmol/L), Uric acid (mg/dL), Creatinine (umol/L), Sodium (mmol/L), Potassium (mmol/L), Glycohemoglobin (%), White blood cells, lymphocytes, monocytes and C-reactive protein, alcohol, Hypercholesterolemia, hypertension, cardiovascular disease, stroke, Cancer, Smoking;

d P < .05 was considered statistically significant.

Table S6. Adjusted Hazard Ratios and 95% CIs of GNRI With Risk of All-Cause Mortality and CVD Mortality for older adults with DN Stratified After Excluding Participants with Hypertension.

| Model | Hazard ratio (95% CI) | | | P value for trend ^d^ |
| --- | --- | --- | --- | --- |
|  | No risk (GNRI＞98) | Low risk (92＜GNRI＜98) | No risk (GNRI＞98) |  |
| All-cause mortality |  |  |  |  |
| Total deaths, No. | 165/441 | 35/441 | 37/441 | NA |
| Model 1^a^ | 1.00 (Reference) | 1.67 (1.16 ~ 2.41) | 1.94 (1.35 ~ 2.77) | <.001 |
| Model 2^b^ | 1.00 (Reference) | 1.91 (1.31 ~ 4.04) | 4.81 (2.08 ~ 11.08) | <.001 |
| Model 3^c^ | 1.00 (Reference) | 3.97 (1.60 ~ 9.88) | 7.05 (1.75 ~ 28.41) | .006 |
| CVD mortality |  |  |  |  |
| Total deaths, No. | 49/441 | 16/441 | 12/441 | NA |
| Model 1^a^ | 1.00 (Reference) | 2.20 (1.25 ~ 3.90) | 1.60 (0.85 ~ 3.03) | .144 |
| Model 2^b^ | 1.00 (Reference) | 3.72 (0.71 ~ 19.53) | 3.90 (0.80 ~ 18.92) | .092 |
| Model 3^c^ | 1.00 (Reference) | 23.55 (0.78 ~ 712.64) | 12.22 (0.53 ~ 279.37) | .117 |

Abbreviations: GNRI, Geriatric Nutrition Risk Index; CVD, cardiovascular disease; DN: diabetic nephropathy; CI: Confidence Interval

a: Crude model;

b: Adjusted for Age, Gender, Race, Education, Marital status, poverty income ratio;

c: Adjust for Age, gender, Race, Education, Marital status, poverty income ratio, alanine aminotransferase (U/L), aspartate aminotransferase (U/L), BUN (mmol L), Cholesterol (mmol/L), Triglycerides (mmol/L), Uric acid (mg/dL), Creatinine (umol/L), Sodium (mmol/L), Potassium (mmol/L), Glycohemoglobin (%), White blood cells, lymphocytes, monocytes and C-reactive protein, alcohol, Hypercholesterolemia, hypertension, cardiovascular disease, stroke, Cancer, Smoking;

d P < .05 was considered statistically significant.

Table S7. Adjusted Hazard Ratios and 95% CIs of GNRI With Risk of All-Cause Mortality and CVD Mortality for older adults with DN Stratified After Excluding Participants with Cardiovascular Disease.

| Model | Hazard ratio (95% CI) | | | P value for trend ^d^ |
| --- | --- | --- | --- | --- |
|  | No risk (GNRI＞98) | Low risk (92＜GNRI＜98) | No risk (GNRI＞98) |  |
| All-cause mortality |  |  |  |  |
| Total deaths, No. | 376/1176 | 74/1176 | 99/1176 | NA |
| Model 1^a^ | 1.00 (Reference) | 1.68 (1.30 ~ 2.16) | 1.86 (1.49 ~ 2.32) | <.001 |
| Model 2^b^ | 1.00 (Reference) | 1.91 (1.23 ~ 2.97) | 1.68 (1.11 ~ 2.53) | .013 |
| Model 3^c^ | 1.00 (Reference) | 1.89 (1.16 ~ 3.07) | 3.15 (1.80 ~ 5.49) | <.001 |
| CVD mortality |  |  |  |  |
| Total deaths, No. | 97/1176 | 17/1176 | 20/1176 | NA |
| Model 1^a^ | 1.00 (Reference) | 1.43 (0.85 ~ 2.41) | 1.37 (0.84 ~ 2.22) | .206 |
| Model 2^b^ | 1.00 (Reference) | 0.61 (0.19 ~ 1.97) | 0.79 (0.27 ~ 2.25) | .653 |
| Model 3^c^ | 1.00 (Reference) | 0.60 (0.17 ~ 2.15) | 1.08 (0.29 ~ 4.01) | .909 |

Abbreviations: GNRI, Geriatric Nutrition Risk Index; CVD, cardiovascular disease; DN: diabetic nephropathy; CI: Confidence Interval

a: Crude model;

b: Adjusted for Age, Gender, Race, Education, Marital status, poverty income ratio;

c: Adjust for Age, gender, Race, Education, Marital status, poverty income ratio, alanine aminotransferase (U/L), aspartate aminotransferase (U/L), BUN (mmol L), Cholesterol (mmol/L), Triglycerides (mmol/L), Uric acid (mg/dL), Creatinine (umol/L), Sodium (mmol/L), Potassium (mmol/L), Glycohemoglobin (%), White blood cells, lymphocytes, monocytes and C-reactive protein, alcohol, Hypercholesterolemia, hypertension, cardiovascular disease, stroke, Cancer, Smoking;

d P < .05 was considered statistically significant.

Table S8. Adjusted Hazard Ratios and 95% CIs of GNRI With Risk of All-Cause Mortality and CVD Mortality for older adults with DN Stratified After Excluding Participants with Stroke.

| Model | Hazard ratio (95% CI) | | | P value for trend ^d^ |
| --- | --- | --- | --- | --- |
|  | No risk (GNRI＞98) | Low risk (92＜GNRI＜98) | No risk (GNRI＞98) |  |
| All-cause mortality |  |  |  |  |
| Total deaths, No. | 507/1528 | 115/1528 | 123/1528 | NA |
| Model 1^a^ | 1.00 (Reference) | 1.79 (1.46 ~ 2.19) | 1.71 (1.41 ~ 2.09) | <.001 |
| Model 2^b^ | 1.00 (Reference) | 1.69 (1.35 ~ 2.11) | 1.72 (1.39 ~ 2.14) | <.001 |
| Model 3^c^ | 1.00 (Reference) | 1.89 (1.29 ~ 2.78) | 2.81 (1.64 ~ 4.82) | <.001 |
| CVD mortality |  |  |  |  |
| Total deaths, No. | 156/1528 | 41/1528 | 27/1528 | NA |
| Model 1^a^ | 1.00 (Reference) | 1.78 (1.26 ~ 2.52) | 1.08 (0.72 ~ 1.63) | .708 |
| Model 2^b^ | 1.00 (Reference) | 1.27 (0.97 ~ 2.77) | 1.22 (0.79 ~ 1.90) | .376 |
| Model 3^c^ | 1.00 (Reference) | 1.08 (0.42 ~ 3.92) | 1.11 (0.26 ~ 4.66) | .889 |

Abbreviations: GNRI, Geriatric Nutrition Risk Index; CVD, cardiovascular disease; DN: diabetic nephropathy; CI: Confidence Interval

a: Crude model;

b: Adjusted for Age, Gender, Race, Education, Marital status, poverty income ratio;

c: Adjust for Age, gender, Race, Education, Marital status, poverty income ratio, alanine aminotransferase (U/L), aspartate aminotransferase (U/L), BUN (mmol L), Cholesterol (mmol/L), Triglycerides (mmol/L), Uric acid (mg/dL), Creatinine (umol/L), Sodium (mmol/L), Potassium (mmol/L), Glycohemoglobin (%), White blood cells, lymphocytes, monocytes and C-reactive protein, alcohol, Hypercholesterolemia, hypertension, cardiovascular disease, stroke, Cancer, Smoking;

d P < .05 was considered statistically significant.

Figure S1. Association between prognostic Geriatric Nutritional Risk Index (GNRI)and all-cause mortality and cardiovascular disease (CVD) mortality in US Elderly Adults with Diabetic Nephropathy Stratified by Age.


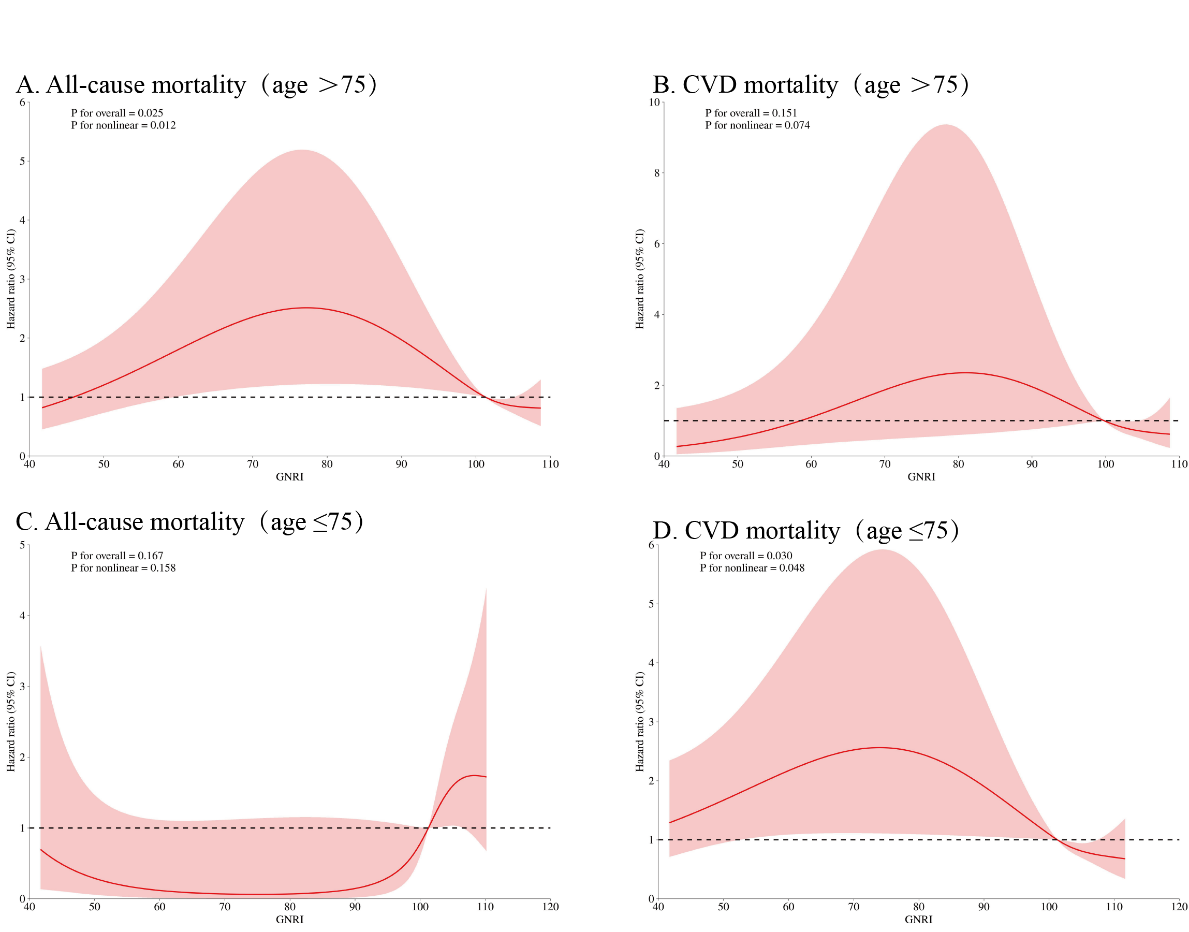


Hazard ratios (solid lines) and 95%CIs (shaded areas) were estimated after adjusting for Age; Sex; Race and ethnicity; Education level; Marital status; Poverty income ratio, Alcohol, Hypertension, Cardiovascular disease, Stroke, Cancer, Smoking.

Figure S2. Association between prognostic Geriatric Nutritional Risk Index (GNRI)and all-cause mortality and cardiovascular disease (CVD) mortality in US Elderly Adults with Diabetic Nephropathy Stratified Stratified by Gender.


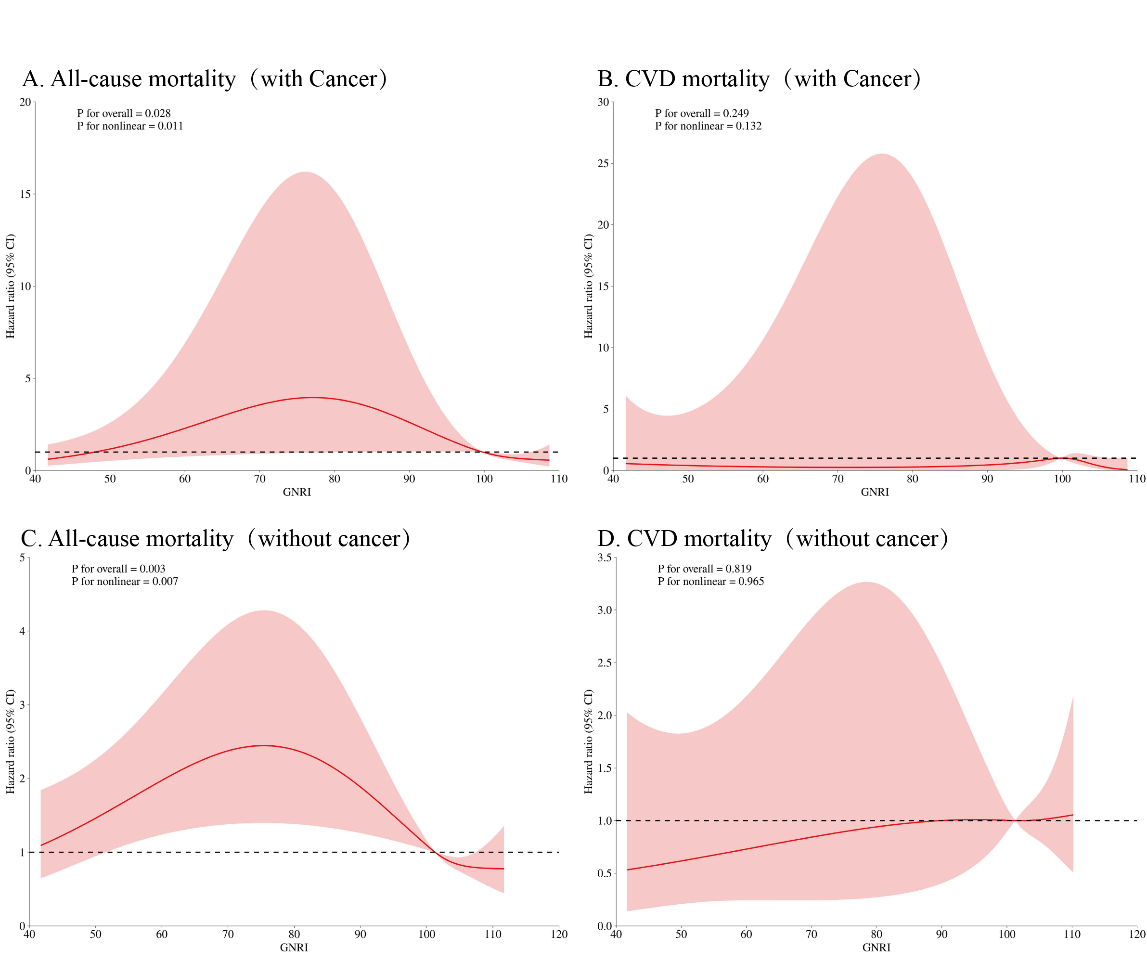


Hazard ratios (solid lines) and 95%CIs (shaded areas) were estimated after adjusting for Age; Sex; Race and ethnicity; Education level; Marital status; Poverty income ratio, Alcohol, Hypertension, Cardiovascular disease, Stroke, Cancer, Smoking.

Figure S3. Association between prognostic Geriatric Nutritional Risk Index (GNRI)and all-cause mortality and cardiovascular disease (CVD) mortality in US Elderly Adults with Diabetic Nephropathy Stratified by Cancer.


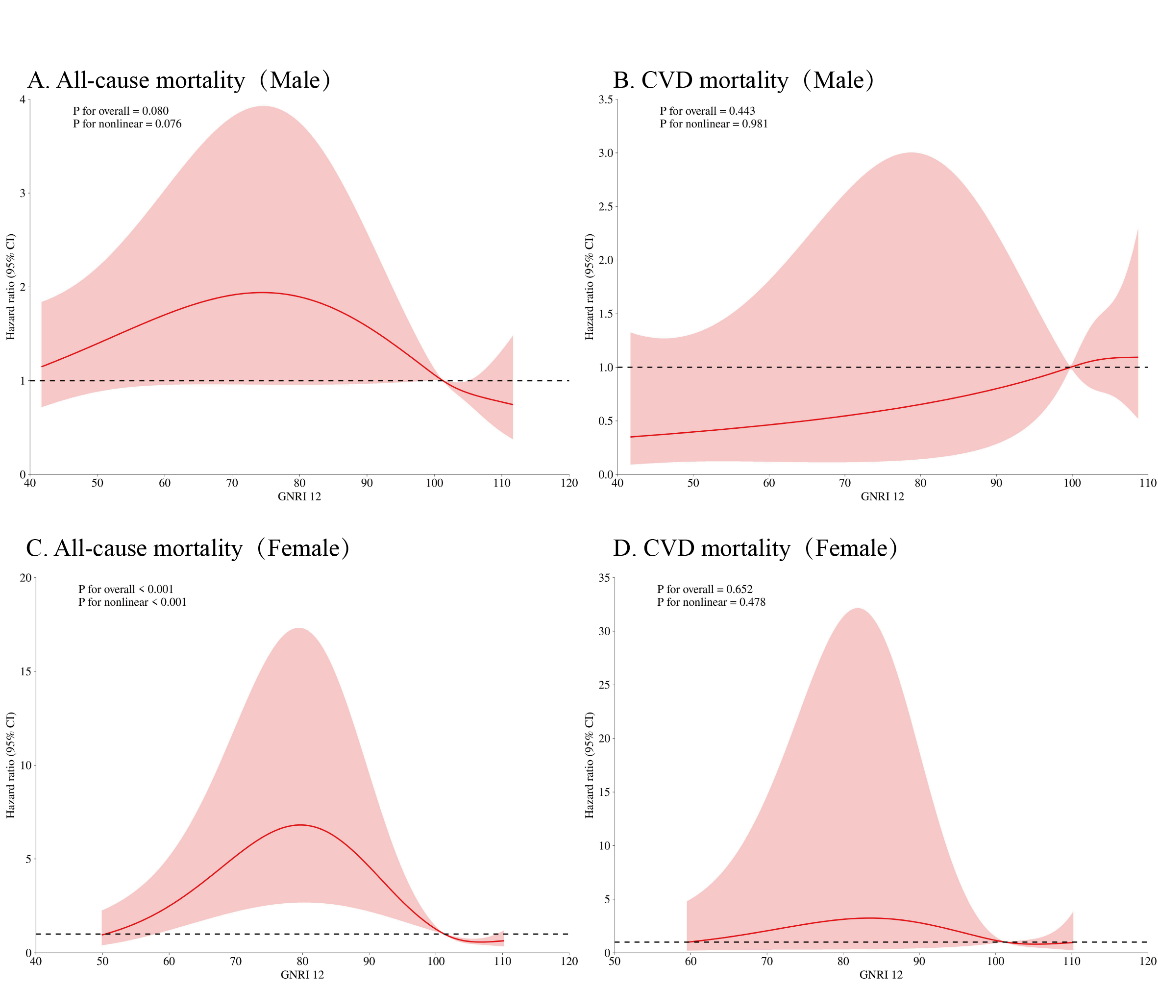


Hazard ratios (solid lines) and 95%CIs (shaded areas) were estimated after adjusting for Age; Sex; Race and ethnicity; Education level; Marital status; Poverty income ratio, Alcohol, Hypertension, Cardiovascular disease, Stroke, Cancer, Smoking.

Figure S4. Association between prognostic Geriatric Nutritional Risk Index (GNRI)and all-cause mortality (A) and cardiovascular disease (CVD) mortality (B) in US Elderly Adults with Diabetic Nephropathy Stratified After Excluding Death Within the First 2 years of Follow-up.


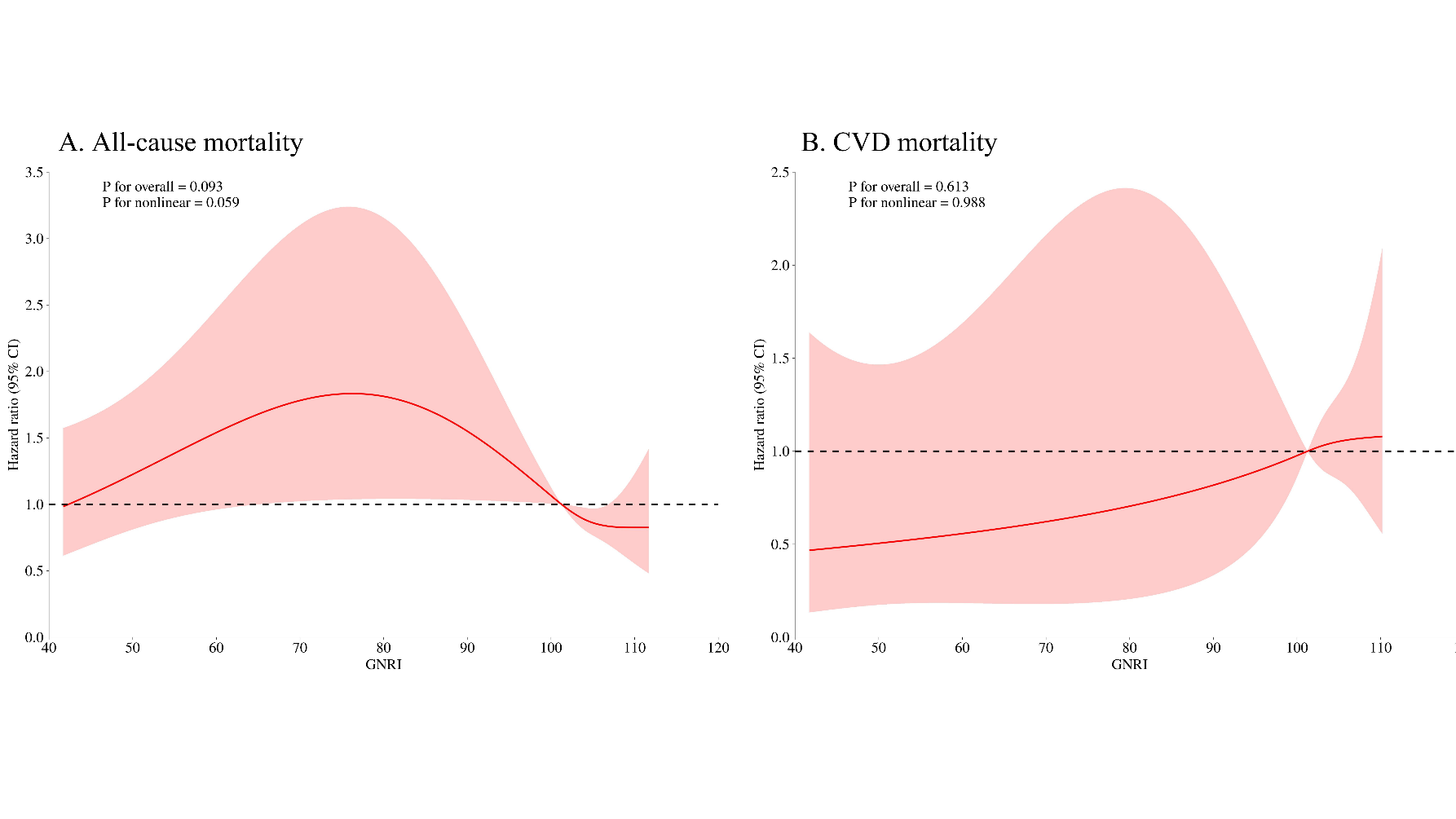


Hazard ratios (solid lines) and 95%CIs (shaded areas) were estimated after adjusting for Age; Sex; Race and ethnicity; Education level; Marital status; Poverty income ratio, Alcohol, Hypertension, Cardiovascular disease, Stroke, Cancer, Smoking.

Figure S5. Association between prognostic Geriatric Nutritional Risk Index (GNRI)and all-cause mortality (A) and cardiovascular disease (CVD) mortality (B) in US Elderly Adults with Diabetic Nephropathy Stratified After Excluding Participants with Hypertension.


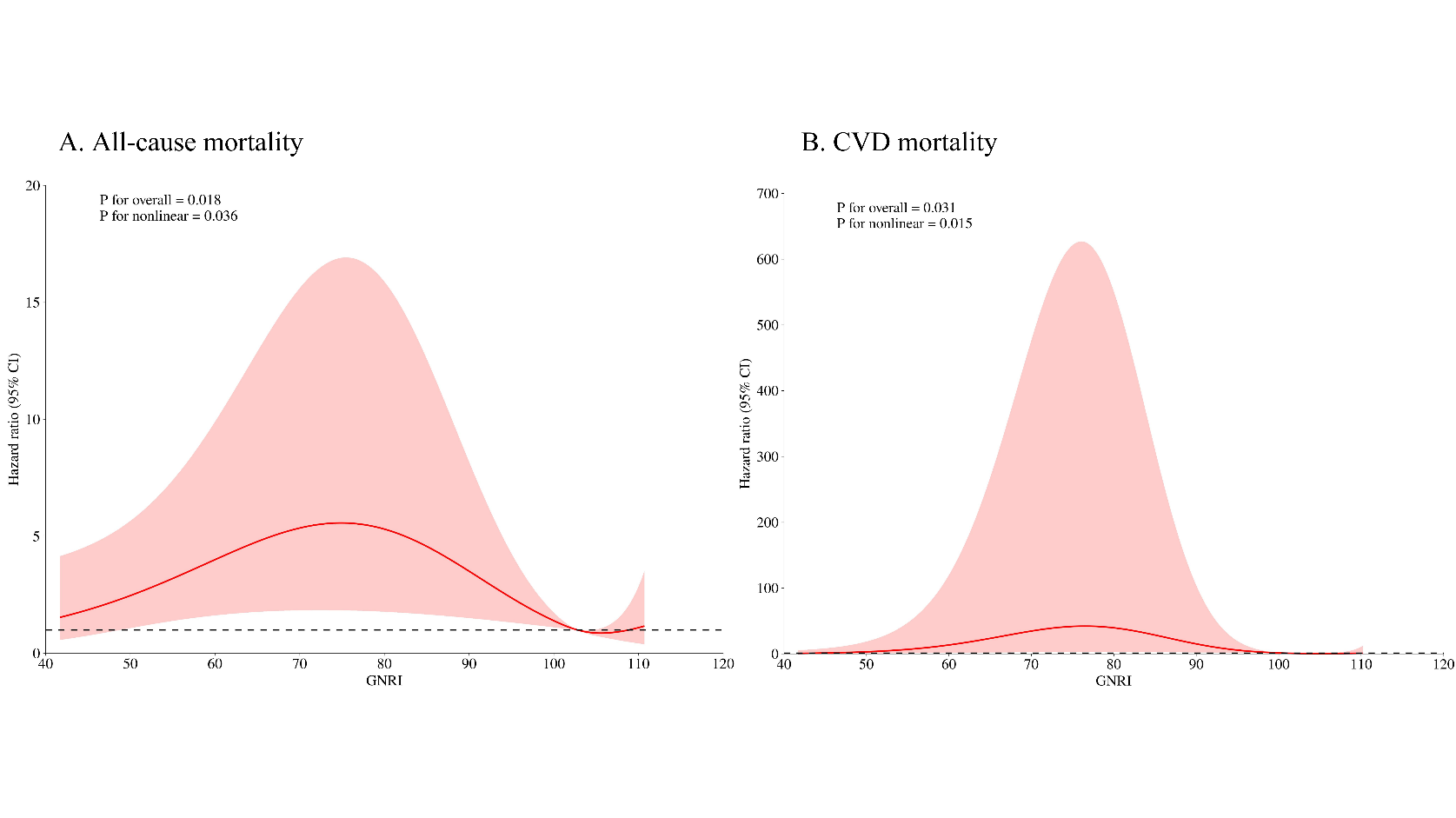


Hazard ratios (solid lines) and 95%CIs (shaded areas) were estimated after adjusting for Age; Sex; Race and ethnicity; Education level; Marital status; Poverty income ratio, Alcohol, Hypertension, Cardiovascular disease, Stroke, Cancer, Smoking.

Figure S6. Association between prognostic Geriatric Nutritional Risk Index (GNRI)and all-cause mortality in US Elderly Adults with Diabetic Nephropathy Stratified After Excluding Participants with Cardiovascular Disease.


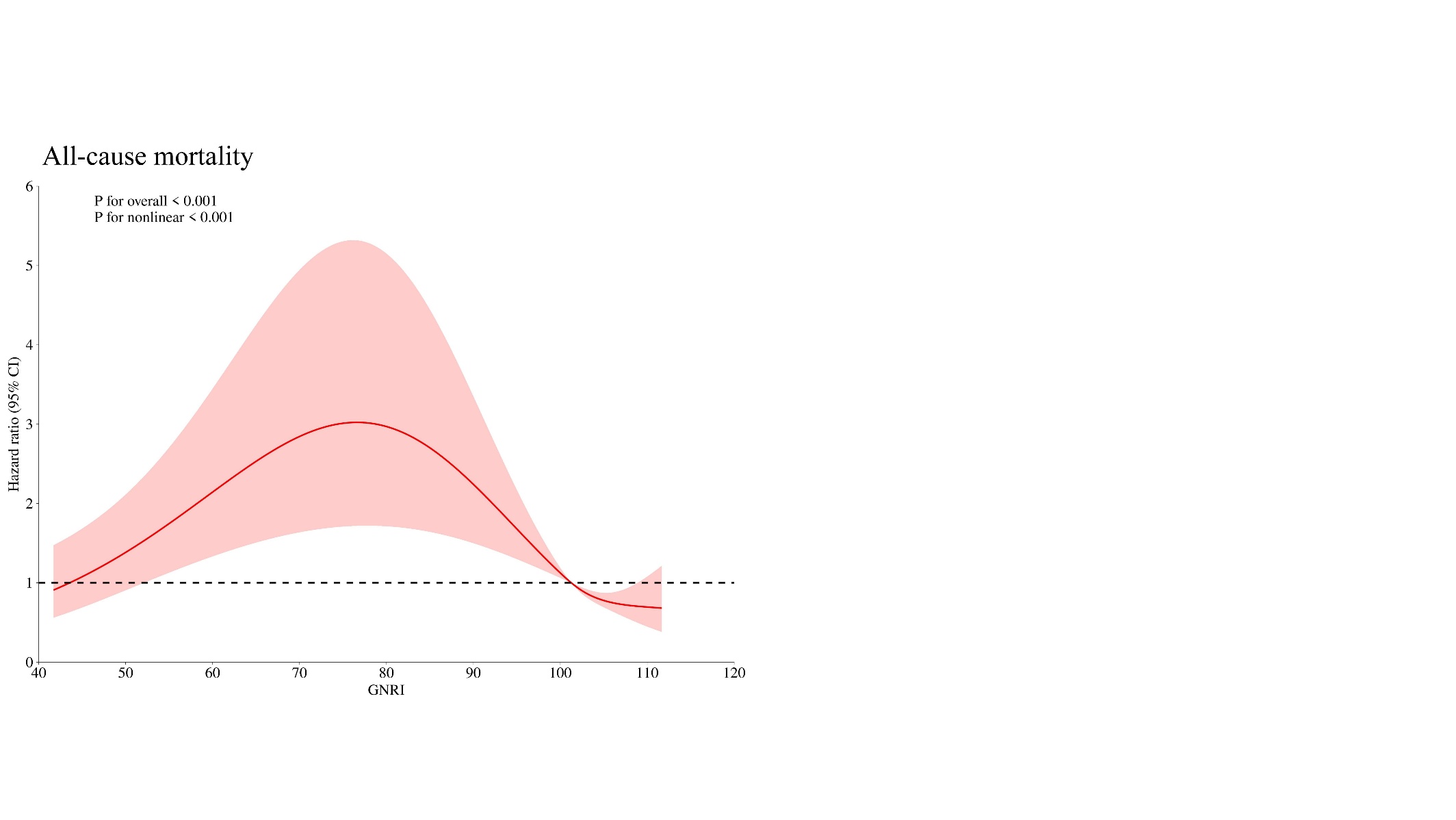


Hazard ratios (solid lines) and 95%CIs (shaded areas) were estimated after adjusting for Age; Sex; Race and ethnicity; Education level; Marital status; Poverty income ratio, Alcohol, Hypertension, Cardiovascular disease, Stroke, Cancer, Smoking.

Figure S7. Association between prognostic Geriatric Nutritional Risk Index (GNRI)and all-cause mortality (A) and cardiovascular disease (CVD) mortality (B) in US Elderly Adults with Diabetic Nephropathy Stratified After Excluding Participants with Stroke.


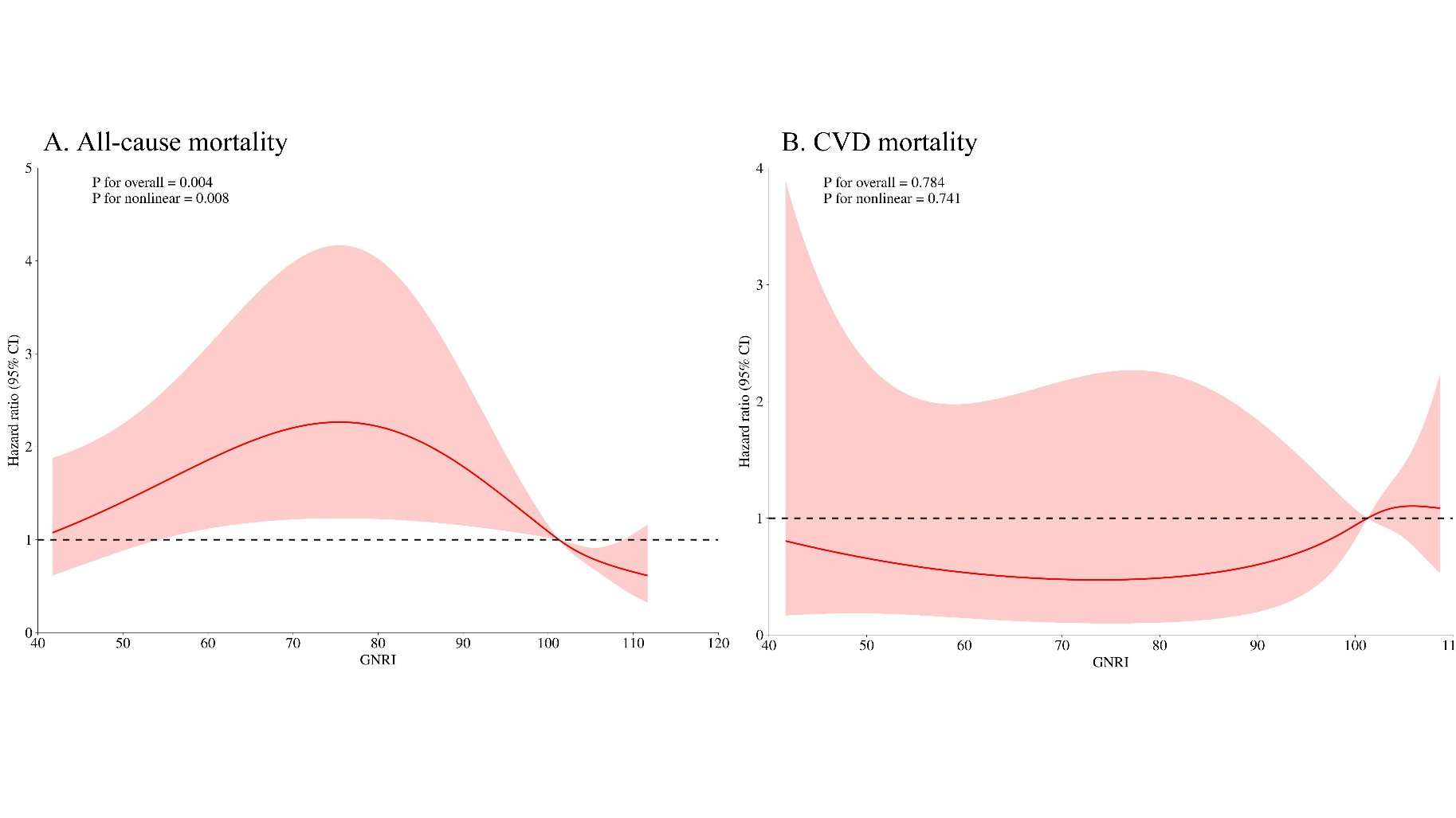


Hazard ratios (solid lines) and 95%CIs (shaded areas) were estimated after adjusting for Age; Sex; Race and ethnicity; Education level; Marital status; Poverty income ratio, Alcohol, Hypertension, Cardiovascular disease, Stroke, Cancer, Smoking.
